# Challenging Isodimorphism Concepts: Formation of Three Crystalline Phases in Poly(hexamethylene-*ran*-octamethylene carbonate) Copolymers

**DOI:** 10.1021/acs.macromol.3c01265

**Published:** 2023-10-11

**Authors:** Yilong Liao, Ricardo A. Pérez-Camargo, Haritz Sardon, Antxon Martínez de Ilarduya, Wenxian Hu, Guoming Liu, Dujin Wang, Alejandro J. Müller

**Affiliations:** † POLYMAT and Department of Polymers and Advanced Materials: Physics, Chemistry, and Technology, Faculty of Chemistry, University of the Basque Country UPV/EHU, Paseo Manuel de Lardizábal, 3, Donostia-San Sebastián 20018, Spain; ‡ Department of Chemical Engineering, Polytechnic University of Catalonia ETSEIB-UPC, Diagonal 647, Barcelona 08028, Spain; § Beijing National Laboratory for Molecular Sciences, CAS Key Laboratory of Engineering Plastics, Institute of Chemistry, Chinese Academy of Sciences, Beijing 100190, P. R. China; ∥ University of Chinese Academy of Sciences, Beijing 100049, P. R. China; ⊥ Ikerbasque, Basque Foundation for Science, Plaza Euskadi 5, Bilbao 48009, Spain

## Abstract

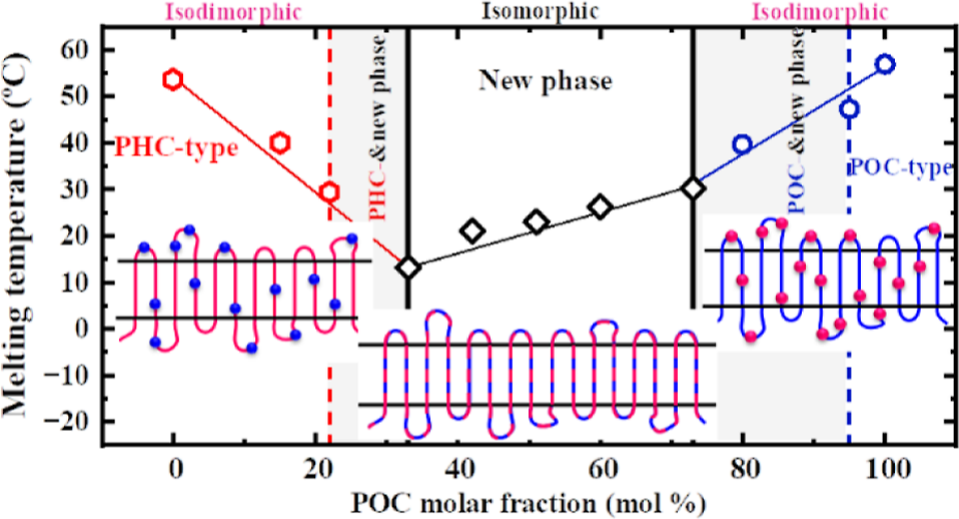

In this work, poly(hexamethylene-*ran*-octamethylene carbonate) copolycarbonates were synthesized by melt polycondensation in a wide range of compositions. The copolymers displayed some of the characteristic isodimorphic thermal behavior, such as crystallization for all the compositions and a pseudoeutectic behavior of the melting temperature (*T*_m_) versus composition. The pseudoeutectic point was located at 33 mol % poly(octamethylene carbonate) (POC) content (i.e., corresponding to the PH_67_O_33_C copolymer). Surprisingly, the crystallinities (*X*_c_) for a wide range of copolymer compositions were higher than those of the parent components, a phenomenon that has not been observed before in isodimorphic random copolymers. The structural characterization, performed by wide-angle X-ray scattering (WAXS) and small-angle X-ray scattering experiments, revealed unexpected results depending on composition. On the one hand, the poly(hexamethylene carbonate) (PHC)- and POC-rich copolymers crystallize in PHC- and POC-type crystals, as expected. Moreover, upon cooling and heating, in situ WAXS experiments evidenced that these materials undergo reversible solid–solid transitions [δ-α (PHC) and δ-α-β (POC)] present in the parent components but at lower temperatures. On the other hand, a novel behavior was found for copolymers with 33–73 mol % POC (including the pseudoeutectic point), which are those with higher crystallinities than the parent components. For these copolymers, a new crystalline phase that is different from that of both homopolymers was observed. The in situ WAXS results for these copolymers confirmed that this novel phase is stable upon cooling and heating and does not show any crystallographic feature of the parent components or their solid–solid transitions. FTIR experiments confirmed this behavior, revealing that the new phase adopts a polyethylene-like chain conformation that differs from the *trans*-dominant ones exhibited by the parent components. This finding challenges the established concepts of isodimorphism and questions whether a combination of crystallization modes (isodimorphism and isomorphism) is possible in the same family of random copolymers just by changing the composition.

## 1 Introduction

Aliphatic polycarbonates (PCs) have been developed as a promising class of biodegradable polymer due to their biocompatibility, biodegradability, nontoxicity, and excellent chemical and physical properties.^1−4^ In particular, they do not generate any acidic compounds upon degradation through surface erosion,^5,6^ which allows them to be suitable for medical applications (e.g., surgical sutures, bone fixation, and drug-controlled release).^3,7^ Moreover, PCs are also intriguing solid polymer electrolytes because of their remarkable ionic conductivity, excellent electrochemical stability, and high lithium transference. This property makes PCs a potential alternative material to poly(ethylene oxide).^8,9^

The advances in synthesis methods allow obtaining PCs without potential toxicity issues from metal catalysts and with a practically unlimited range of chain lengths, *n*_CH2_.^4,10−12^ The use of the polycondensation between diols and dimethyl carbonate opened the possibility of creating PCs with a wide range of *n*_CH2_ and permitted studying the even–odd effect on PCs with *n*_CH2_ = 6 to 12.^13^ Such advances in the synthesis methods allow the preparation of copolycarbonates that combine comonomers (i.e., PCs) with different *n*_CH2_, aiming to widen the range of properties of these materials. Copolymerization offers a better modification over the structures than blends to achieve tunable properties matching various applications.^14−17^ Random covalent links ensure the miscibility of the material obtained from different comonomers that might otherwise be immiscible. This allows for the adjustment of the crystallinity degrees and tailoring of biodegradation rates by introducing a comonomer and varying the composition.

The crystallization behavior of random copolymers is an exciting topic since they can crystallize in three different crystallization modes: isomorphism, isodimorphism, and comonomer exclusion or no-crystallization mode.^18^ All of these modes depend on the comonomer exclusion/inclusion balance.

Isomorphic copolymers are characterized by total comonomer inclusion within the crystal lattice (i.e., cocrystallization), which occurs under strict molecular features,^19−21^ e.g., similar chemical structures of the repeating units. This total comonomer inclusion is evidenced by the crystallization in all of the compositions, exhibiting thermal and structural properties that show a linear dependence on the composition. On the contrary, when total comonomer exclusion dominates, the resulting random copolymers can only crystallize in a limited composition range, as even smaller comonomer contents can limit the crystallization of the second comonomer-rich phase.

Isodimorphism is an intermediate case between the crystallization modes mentioned above and can be viewed as its combination. In isodimorphic copolymers, a competition between inclusion and exclusion occurs, where typically inclusion can only be tolerated up to a certain degree that depends on the chemical affinity of the comonomers. Comonomer exclusion hinders the crystallization of the major phase, provoking the depression of different physical properties, e.g., crystallinity degree, crystallization (*T*_c_), and melting (*T*_m_) temperatures. Partial comonomer inclusion, as evidenced by the changes in the major crystalline phase, e.g., different interplanar distances within the lattice unit cell of the copolymers compared with the parent components, avoids the complete depression of these properties, allowing crystallization for all of the compositions. This particular comonomer exclusion/inclusion balance provokes a pseudoeutectic behavior of the thermal transitions, e.g., *T*_m_, as a function of the comonomer content. Considering A_*x*_B_*y*_ copolymers, on one side of the pseudoeutectic point, where the material typically exhibits minimum values of *T*_c_ and *T*_m_, the material crystallizes in the A-rich crystalline phase with the inclusion of some percentage of B counits. On the opposite side, the material crystallizes in the B-rich crystalline phase with the inclusion of some percentage of A counits. In our previous findings, we observed the coexistence of both A-rich and B-rich crystalline phases at the pseudoeutectic point, as detected by wide-angle X-ray scattering (WAXS) experiments or double melting (sequential melting of each phase) detected by differential scanning calorimetry (DSC).^15,22−25^

The chemical demands of isodimorphism are not as strict as those of isomorphism; thus, isodimorphic behavior has been widely reported for several random copolymer systems. In particular, isodimorphism in copolyesters has been thoroughly studied.^15,19,22,23,26−28^ In the case of copolycarbonates, a few systems have been prepared to combine aliphatic PCs comonomers with different *n*_CH2_ (PC*n*_CH2_). Zhu et al.^29^ studied PC4-*ran*-PC6 copolycarbonates, determining that cocrystallization cannot occur, thus limiting the crystallization to the richest compositions in one or another comonomer, i.e., 10:90 and 90:10 PC4-*ran*-PC6. Similar results were reported by Arandia et al.^15^ in PC4-*ran*-PC7 copolycarbonates in which only compositions with higher contents than 80 mol % PC4 or 80 mol % PC7 can crystallize. These authors explained that rather than comonomer exclusion, which occurs when there is a significant disparity in the chemical structures of the two repeating units, the observed behavior is provoked by the slow crystallization kinetics of both comonomers. It was argued that under the right conditions, e.g., extremely slow cooling, these copolycarbonates should display isodimorphic behavior.

The isodimorphic behavior in copolycarbonates is more apparent when PC4 is combined with *n*_CH2_ > 7 comonomers. Zhang et al.^11^ found that PC4-*ran*-PC10 copolycarbonates crystallize for all compositions showing a pseudoeutectic behavior with the pseudoeutectic point at 20 mol % of PC10. Therefore, only the richest composition in PC4, i.e., 90:10 PC4-*ran*-PC10, can crystallize with a PC4 crystalline structure.

Arandia et al.^15^ reported isodimorphic behavior in PC4-*ran*-PC12 copolycarbonates. These copolycarbonates crystallize for all compositions, displaying a pseudoeutectic point at 15 mol % of PC12 content. At the pseudoeutectic point, a double melting transition was found due to PC4-rich phase crystals melting at low temperatures, followed by PC12-rich phase crystals melting. Besides the PC4-based combinations, they also studied the PC7-*ran*-PC12 copolycarbonates, which are also isodimorphic, with a pseudoeutectic point at 20 mol % of PC12 content. The 80:20 PC7-*ran*-PC12 copolycarbonate displayed a novel behavior since the nonisothermal DSC scans revealed both coincident crystallization and melting despite being a double crystalline structure containing PC7- and PC12-rich crystalline phases detected by WAXS. Recently, Hung et al.^27^ studied PC8-*ran*-PC10 copolycarbonates. These authors found that these materials are isodimorphic with a pseudoeutectic region between 21 and 36 mol % of PC10 content. In addition, they studied the solid–solid transition for those compositions with PC8 content ≥79 mol %.

Poly(octamethylene carbonate) (PC8 or POC) exhibits a solid–solid crystalline transition. For POC, Zhao et al.^10^ first reported a reversible α-β solid phase transition, which is analogous to the Brill transition observed in polyamides.^30,31^ It was primarily related to the reversible conformational change in methylene sequences, transitioning from a predominantly *trans* configuration at low temperatures to a coexistence of *trans/gauche* conformations at high temperatures. Pérez-Camargo et al.^32,33^ disclosed a new reversible α-δ solid phase transition that occurs at a lower temperature than the α-β phase transition. The δ phase is characterized by more efficient packing of methylene groups and ordered chain conformations in comparison to the α phase. A reversible δ-α solid–solid phase transition has been disclosed in poly(hexamethylene carbonate) (PHC).^32,33^

This work represents the first contribution to the study of a new series of aliphatic random copolycarbonates based on POC: poly(hexamethylene-*ran*-octamethylene carbonate) (PH_*x*_O_*y*_C), poly(heptamethylene-*ran*-octamethylene carbonate), and poly(octamethylene-*ran*-dodecamethylene carbonate) copolycarbonates. Here, we focus on PH_*x*_O_*y*_C copolycarbonates synthesized using 4-dimethylaminopyridine (DMAP) as an organocatalyst in a two-step polycondensation process. The thermal properties, structure, conformation, and morphology of the PH_*x*_O_*y*_C copolymers were investigated in detail. The DSC results demonstrate the isodimorphic-like behavior in these copolymers. Structural characterization shows that PHC- and POC-rich copolymers follow an isodimorphic-like behavior, crystallizing in PHC- or POC-type crystals, including the solid–solid transitions of these materials. But a novel and unique behavior was found for a wide range of compositions. A new stable crystalline phase was observed, similar to those observed in some isomorphic copolymers, without any trace of the crystalline structure of the parent components, not even their solid–solid transitions, instead of the typical mixture of crystalline phases that are found in other isodimorphic copolycarbonates. FTIR confirmed the presence of this new stable phase, which adopts a PE-like chain conformation, differing from the *trans*-dominant conformations of the parent homopolymers. This finding challenges the isodimorphism concepts and leads us to ask whether a combined isomorphism–isodimorphism crystallization is possible.

## 2 Experimental Part

### 2.1 Materials

Dry dimethyl carbonate (DMC) (99+ %) and DMAP (99%) were purchased from Across Organics. Sigma-Aldrich supplied 1,6-hexanediol (99+ %) and 1,8-octanediol (99+ %). Before use, the two diols and DMAP were dried for 5 h. Chloroform (99+ %) and methanol (MeOH) (certified AR for Analysis) were purchased from Fisher Scientific. Tetrahydrofuran (THF) [size exclusion chromatography (SEC) grade] was obtained from Scharlab, toluene (HPLC grade) from Sigma-Aldrich, and deuterated chloroform (99.8%) (CDCl_3_) from Deutero GmbH. The chemical route for synthesizing copolycarbonates via the melt polycondensation method has been reported in our previous work.^9,15^

### 2.2 Chemical Structure Characterization (NMR and SEC)

The ^1^H and ^13^C nuclear magnetic resonance (NMR) tests were carried out on Bruker AVANCE NEO 500 spectrometers at room temperature, with CDCl_3_ used as the solvent. We calibrated the spectra using the CDCl_3_ peak (δ_H_ = 7.26 ppm and δ_C_ = 77.16 ppm).

The number-average (*M*_n_) and weight-average (*M*_w_) molecular weights, as well as the dispersity (*D̵*), of the synthesized materials were measured by using SEC. The equipment utilized for this purpose consisted of three columns in series (Styragel HR2, HR4, and HR6, with pore sizes ranging from 1 × 10^2^ to 1 × 10^6^ Å), an LC20 pump (Shimadzu) coupled to a DAWN Heleos multiangle light scattering laser photometer, and a differential refractometer (all from Wyatt Technology Corp., USA). The sample was dissolved in THF (SEC grade) at a concentration of 5 mg/mL, and the analyses were performed at 35 °C using THF as a mobile phase at a flow rate of 1 mL/min. Narrow polystyrene standards were used for calibration.

### 2.3 Differential Scanning Calorimetry

This study used a PerkinElmer 8500 calorimeter with a controlled liquid nitrogen cooling system, model CLN2, to investigate homopolymers’ and copolymers’ nonisothermal crystallization and melting behavior. The instrument calibration was achieved by using high-purity indium and tin standards. Samples weighing around 5 mg were encapsulated in standard aluminum DSC pans and subjected to DSC experiments in an ultrapure nitrogen atmosphere with a flow rate of 20 mL/min. The DSC experiments were conducted at a scan rate of 10 °C/min over a temperature range of −40–100 °C. Thermal history was erased by heating the samples to 100 °C for 3 min and then cooling down to −40 °C for 1 min to crystallize. Finally, a heating scan was conducted to record the melting endotherms.

### 2.4 Small-Angle and Wide-Angle X-ray Scattering

Simultaneous small-angle X-ray scattering (SAXS)/WAXS data were obtained at the 1W2A beamline (λ = 1.54 Å) of the Beijing Synchrotron Radiation Facility (BSRF). The WAXS patterns were recorded using a MAR165 CCD detector, with a resolution of 2048 × 2048 pixels (pixel size: 79 μm × 79 μm), and the sample-to-detector distance was 263.9 mm with a tilt angle of 15°. In the SAXS configuration, a Pilatus 1 M detector, with a resolution of 981 × 1043 pixels (pixel size: 172 μm × 172 μm), was employed. The sample-to-detector distance was 3202 mm. One-dimensional intensity profiles were integrated using the standard procedure with the substrate for background and reported as the scattering intensity versus scattering vector, *q* = 4π sin θ/λ, in which 2θ is the Bragg angle.

For nonisothermal measurements, samples were encapsulated in a DSC aluminum pan and placed in a Linkam THMS600 hot stage coupled to a liquid nitrogen cooling system. Simultaneous WAXS/SAXS diffractograms were recorded every 2 °C as the polymers underwent crystallization and melting. The cooling and heating conditions employed in the nonisothermal DSC experiments were replicated, ensuring that the results obtained from WAXS/SAXS were comparable.

### 2.5 Fourier Transform Infrared Spectroscopy

The variable-temperature infrared spectra were recorded by a Nicolet 6700 FTIR spectrometer from Thermo Fisher connected to a Linkam FTIR600 hot-stage. The resolution of spectra was 4.0 cm^–1^, and the acquisition time of each spectrum was 30 s. The samples for FTIR measurements were drop-cast onto potassium bromide (KBr) plates from a solution in chloroform with a concentration of 5 mg/mL. The same thermal protocol described in Section 2.3 was applied.

### 2.6 Polarized Light Optical Microscopy

The morphology of the sample was studied using an Olympus BX51 polarized light optical microscope equipped with a λ plate inserted at 45° with respect to the polarization direction. Images were captured using an Olympus SC50 digital camera, and precise temperature control was achieved with a THMS600 Linkam hot stage connected to liquid nitrogen. The samples were melted on a glass slide covered with a thin glass coverslip and crystallized from the melt. First, the thermal history was erased for 3 min at 100 °C. Next, the samples were cooled to −40 °C at 1 °C/min. The micrographs were taken at −40 °C.

## 3 Results and Discussion

### 3.1 Synthesis and Chemical Characterization

**Scheme 1:**
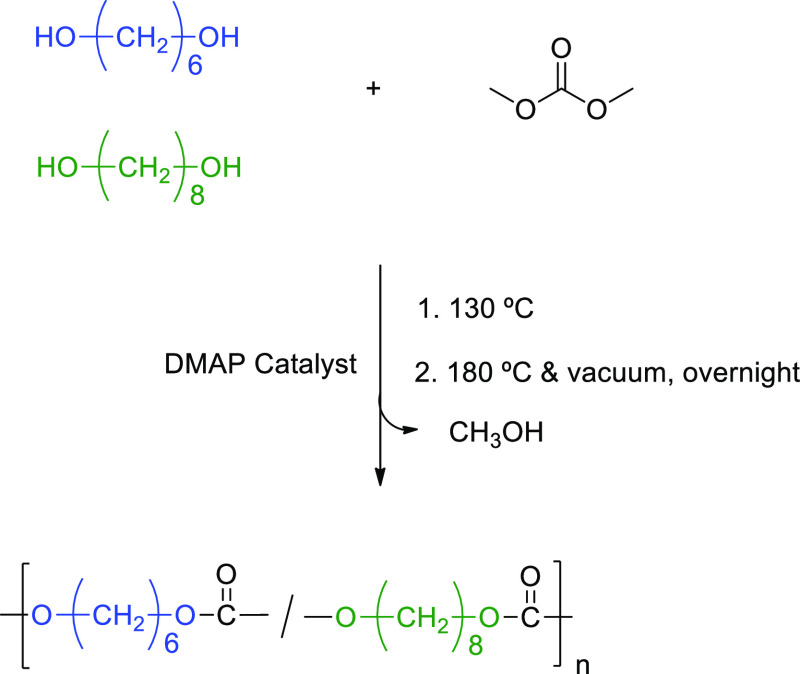
Synthesis by Polycondensation of Diols with Dimethyl Carbonate of Aliphatic Polycarbonate Copolymers

We employed a melt polycondensation synthetic protocol, as outlined in Scheme 1, to synthesize PH_*x*_O_*y*_C copolymers in a wide range of compositions. The monomers, 1,6-hexanediol, and 1,8-octanediol, were coreacted with DMC catalyzed by DMAP. The syntheses were conducted in a 50 mL round-bottom flask connected to a vacuum. During the initial step, the dried reagents, including DMC, diols, and the organocatalyst DMAP, were added to the flask and placed in an oil bath at 130 °C for 4 h. The temperature was then raised to 180 °C, and a high vacuum was applied overnight to remove the reaction byproduct. The high temperature and vacuum conditions were critical for increasing the molar masses. To purify the polymers, we dissolved the obtained samples in chloroform and then precipitated them in cold methanol. A 2:1:0.01 molar ratio of DMC/diol/DMAP was used for all reactions. By adjusting the feed contents of 1,6-hexanediol and 1,8-octanediol, PH_*x*_O_*y*_Cs were synthesized.

**Figure 1:**
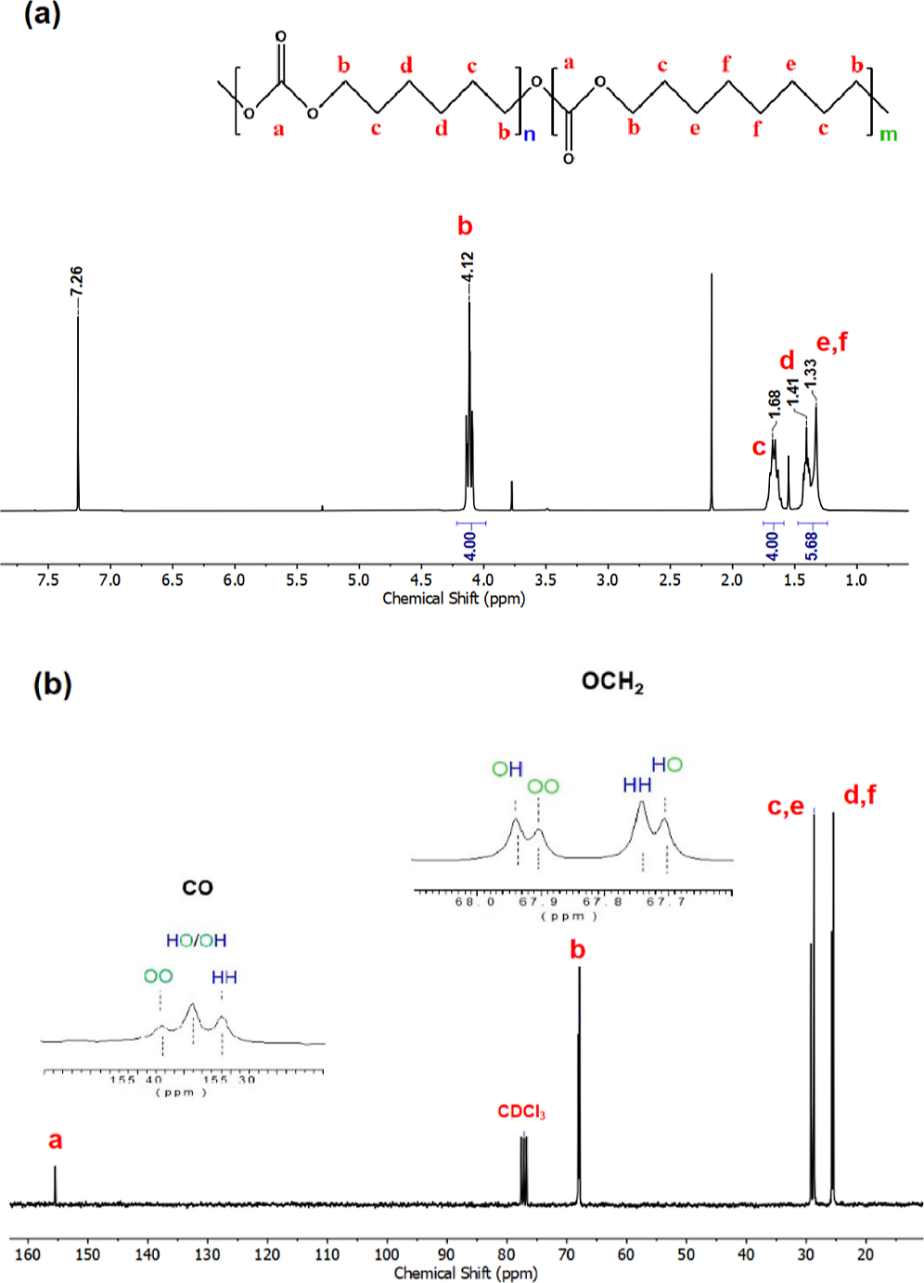
(a) ^1^H and (b) ^13^C NMR spectra of copolymer PH_58_O_42_C in CDCl_3_. The peaks’ assignment is marked with red letters in the chemical structure.

The chemical structures of the copolymers were first analyzed by NMR. Figure 1 provides the ^1^H NMR and ^13^C NMR spectra of a representative copolymer with the initial monomer feed of 60:40 (hexanediol/octanediol). The remaining copolymers were also analyzed by ^1^H and ^13^C NMR, and the corresponding spectra are provided in Figures S1 and S2.

The composition of the resulting copolymer can be quantified by utilizing the integral intensities of the characteristic signals in the ^1^H NMR spectrum. The chemical shifts and peak assignments are as follows: δ = 4.12 ppm (t, OCOOC***H***_**2**_, 4H·(*x*_PHC_ + *x*_POC_)), 1.68 ppm (q, OCOOCH_2_C***H***_**2**_, 4H·(*x*_PHC_ + *x*_POC_)), 1.41 ppm (t, OCOOCH_2_CH_2_C***H***_**2**_C***H***_**2**_CH_2_CH_2_OCOO, 4H·*x*_PHC_ (PHC)), and 1.33 ppm (m, OCOOCH_2_CH_2_C***H***_**2**_C***H***_**2**_C***H***_**2**_C***H***_**2**_CH_2_CH_2_OCOO, 8H·*x*_POC_ (POC)). A triplet between 4.0 and 4.15 ppm is ascribed to the resonance peaks of methylene protons adjacent to the oxygen atom of the carbonate group (4·*x*_PHC_ for PHC and 4·*x*_POC_ for POC), which integrates 4 protons. The integrated area of the rest of the peaks was thus obtained with respect to this area. Specifically, the signal peaks at 1.41 and 1.33 ppm, assigned to the inner methylenes of PHC and POC repeating units, respectively, have an integration value of 5.68. This value comprises 4·*x*_PHC_ protons from PHC and 8·*x*_POC_ protons from POC. Based on these findings, two equations were used to estimate the molar ratio of repeating units (*x*_POC_ and *x*_PHC_)
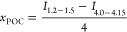
1
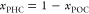
2where *I*_4.0–4.15_ denotes an integral area between 4.0 and 4.15 ppm, corresponding to those protons assigned to the methylenes close to the oxygen atoms, while *I*_1.2–1.5_ represents the area between 1.2 and 1.5 ppm, representing those inner methylenes of the PHC and POC repeating units. Therefore, the molar ratio of repetitive units in this copolymer was calculated to be around 58 mol % PHC and 42 mol % POC, and thereby, the sample was named PH_58_O_42_C based on the calculated molar ratio of PHC and POC in the copolymer. The final compositions of other copolymers were determined by using the same methodology, and the results are listed in Table 1. Although the calculated compositions are not identical to the feed ratio, the two values are very close.

**Table 1:** Composition and Microstructure of PH_*x*_O_*y*_C Copolymers and Parent Homopolymers

copolycarbonates	composition (mol %)^a^		dyad content (mol %)^b^			sequence length		*R*	*M*_w_ (kg/mol)^c^	*D̵*^c^
	*X*_PHC_	*X*_POC_	HH	HO/OH	OO	*N*_HC_	*N*_OC_			
PHC	100.0	0	100.0	0	0				28	1.92
PH_85_O_15_C	84.7	15.3	78.7	19.0	2.3	9.2	1.2	0.9	8	1.92
PH_78_O_22_C	77.9	22.1	60.5	32.4	7.1	4.7	1.4	0.9	19	2.28
PH_67_O_33_C	67.2	32.8	44.0	41.7	14.3	3.1	1.7	0.9	14	2.10
PH_58_O_42_C	58.0	42.0	35.9	48.5	15.7	2.5	1.7	1.0	15	2.39
PH_49_O_51_C	49.1	50.9	25.0	51.0	24.0	2.0	1.9	1.0	16	2.10
PH_40_O_60_C	39.5	60.5	14.5	47.0	38.5	1.6	2.6	1.0	10	1.86
PH_27_O_73_C	27.0	73.0	10.5	45.1	44.4	1.5	3.0	1.0	22	2.19
PH_20_O_80_C	20.0	80.0	3.6	35.5	60.9	1.2	4.4	1.0	16	2.11
PH_5_O_95_C	5.0	95.0	0.4	20.3	79.3	1.0	8.8	1.1	13	2.21
POC	0	100.0	0	0	100.0				14	2.05

a Compositions of copolymers were calculated by^1^H NMR, and the copolymers were named PH_*x*_O_*y*_C, where the subscripts of *x* and *y* represent the molar percent (as integers) of PHC and POC, respectively.

b The sequence distributions of HH, HO/OH, and OO dyads were calculated based on the intensity ratio of the signals appearing around 67.8 ppm in the^13^C NMR spectra. The degree of randomness was determined from the average sequence lengths.

c The weight-average molecular weight *M*_w_ and dispersity *D̵* were determined through SEC.

The distribution of the two comonomers plays a crucial role in determining the final properties of copolymers. The sensitivity of ^13^C NMR to the difference of carbon atoms within different chemical environments can be used to obtain valuable information on the distribution of the repeating units along the backbone and the degree of randomness.^34^ To this end, Figure 1b presents the ^13^C NMR spectrum of copolymer PH_58_O_42_C, and a precise assignment of the characteristic peaks was made along with the NMR spectrum to determine its microstructure. The signals around 155.3 and 67.8 ppm split into three and four peaks, respectively, and were assigned to carbonyl and -O**C**H_2_ carbon resonance. The three peaks located at 155.31, 155.34, and 155.37 ppm were further attributed to different dyad sequence distributions: HH (PHC-PHC), HO (PHC-POC), OH (POC-PHC), and OO (POC-POC), respectively. Additionally, the four split peaks at 67.70, 67.74, 67.90, and 67.94 ppm were assigned to the HO, HH, OO, and OH dyads. In this way, the degree of randomness (*R*) could be calculated with the –O**C**H_2_ carbon resonance signals at around 67.7 ppm using eqs 3–5
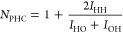
3
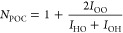
4
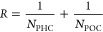
5where *I*_HH_, *I*_HO_, *I*_OH_, and *I*_OH_ are the integration of the –O**C**H_2_ carbon resonance signals of HH, HO, OH, and OO, respectively. *N*_PHC_ and *N*_POC_ represent the number-average sequence length of the PHC and POC repetitive units, respectively. Regarding copolymer microstructures, *R* = 1 indicates a random copolymer, while *R* = 2 means the copolymer is alternating, and in a block copolymer, *R* ∼ 0. As seen in Figure S3, similar NMR analyses were conducted on all copolymers, and using eqs 3–5, the degrees of randomness thus were obtained. As summarized in Table 1, the *R* values are close to 1, indicating a random microstructure of the synthesized PH_*x*_O_*y*_C copolymers.

Molecular weight is another important factor affecting the properties of the materials. The molecular weights and dispersity were determined by SEC. As shown in Figure S4 and Table 1, the *M*_w_ of the polymers obtained falls mostly in the range of 13,000–28,000 g/mol, with dispersity of 1.80–2.40, which are common values for polycondensation polymers. These results demonstrate a comparable level, which facilitates further exploration.

### 3.2 Nonisothermal DSC Experiments

**Figure 2:**
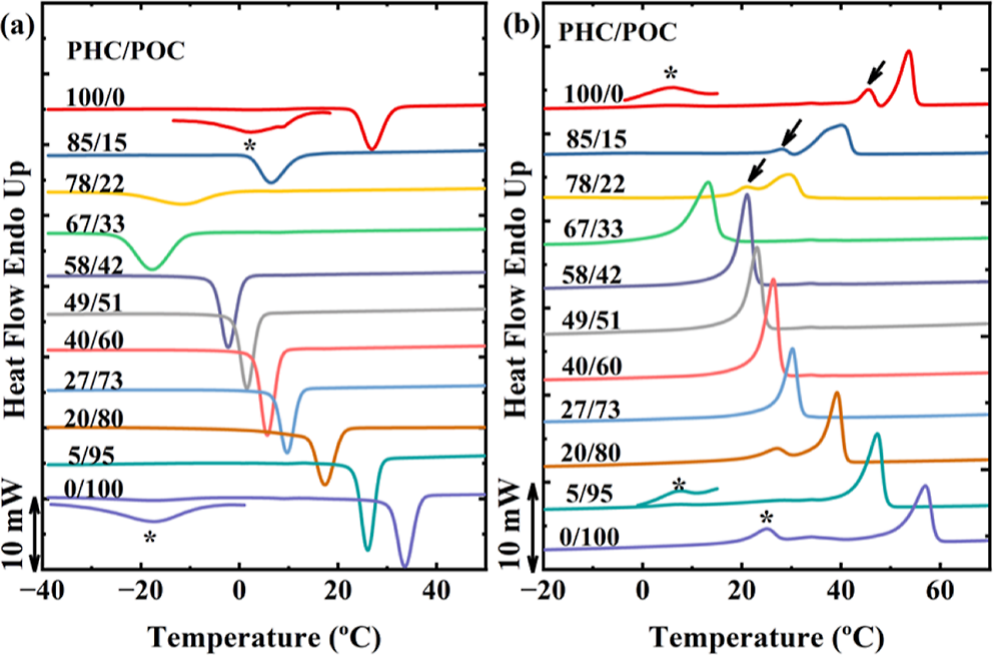
DSC (a) cooling scan from the isotropic melt and (b) subsequent heating scans for all materials. Scanning rates: 10 °C/min. The enlarged signals marked with an asterisk (*) correspond to the reversible α-δ solid–solid phase transition during cooling and heating scans. The arrows indicate the melt-recrystallization behavior.

The thermal properties of PHC and POC homopolymers and PH_*x*_O_*y*_C copolymers were examined by nonisothermal DSC scans. Figure 2 displays the standard nonisothermal cooling (Figure 2a) and second heating (Figure 2b) DSC scans, and all relevant transition temperatures and enthalpies are listed in Table 2.

**Table 2:** Thermal Properties Obtained by DSC for PHC, POC, and PH_*x*_O_*y*_C Copolymers

sample	*T*_c_ (°C)	*T*_m_ (°C)	*T*_m,end_ (°C)	Δ*H*_m_ (J/g)^a^	*X*_c,WAXS_ (%)^b^
PHC	26.9	45.5/53.6	47.2/55.1	40	47.3
PH_85_O_15_C	6.5	28.1/40.0	30.0/42.5	35	46.3
PH_78_O_22_C	–11.5	20.7/29.6	23.2/32.3	27	43.9
PH_67_O_33_C	–17.8	13.2	15.4	40	47.1
PH_58_O_42_C	–2.3	21.0	22.7	41	50.3
PH_49_O_51_C	1.5	23.0	24.7	43	51.8
PH_40_O_60_C	5.6	26.3	28.0	47	55.3
PH_27_O_73_C	9.7	30.2	32.0	44	53.1
PH_20_O_80_C	17.3	27.0/39.2	29.6/41.8	43	53.4
PH_5_O_95_C	26.0	47.3	49.0	40	51.1
POC	33.6	25.0/34.1/56.9	27.8/41.6/59.0	43	48.5

a Melting enthalpy (Δ*H*_m_) obtained using DSC at 10 °C/min.

b The degree of crystallinity (*X*_c,WAXS_) for all samples was calculated based on the results of peak fitting to WAXS curves (see Figure S7).

As reported in previous papers,^9,13^ POC crystallizes and melts at higher temperatures than PHC. Both parent components show (see an asterisk in Figure 2) exothermic and endothermic signals that correspond to the α-δ and δ-α reversible solid–solid phase transition, respectively.^10,32,33^ In POC, a shoulder in between the δ-α transition and the melting peak (*T*_m_) corresponds to the reported α-β transition.^10,32^ This transition is easier to detect by WAXS experiments (see Section 3.3). Figure 2b shows the δ-α transition for PH_5_O_95_C at lower values than for the POC. Details on how this transition is influenced by copolymerization are presented below.

Figure 2 shows that despite the random comonomer distribution (confirmed previously by ^13^C NMR), all PH_*x*_O_*y*_C copolymers can crystallize during cooling (Figure 2a), displaying a single crystallization peak (*T*_c_). The DSC heating curves are plotted in Figure 2b. It can be seen that *T*_c_ and *T*_m_ of the copolymers are located below those exhibited by the parent components. Both exo- and endothermic signals are broader for PHC-rich compositions and sharper for POC-rich and intermediate compositions. The neat PHC and its PHC-rich copolymers (PH_85_O_15_C and PH_78_O_22_C) display an extra endothermic signal, corresponding to melting recrystallization behavior similar to the PHC.^32,35^

**Figure 3:**
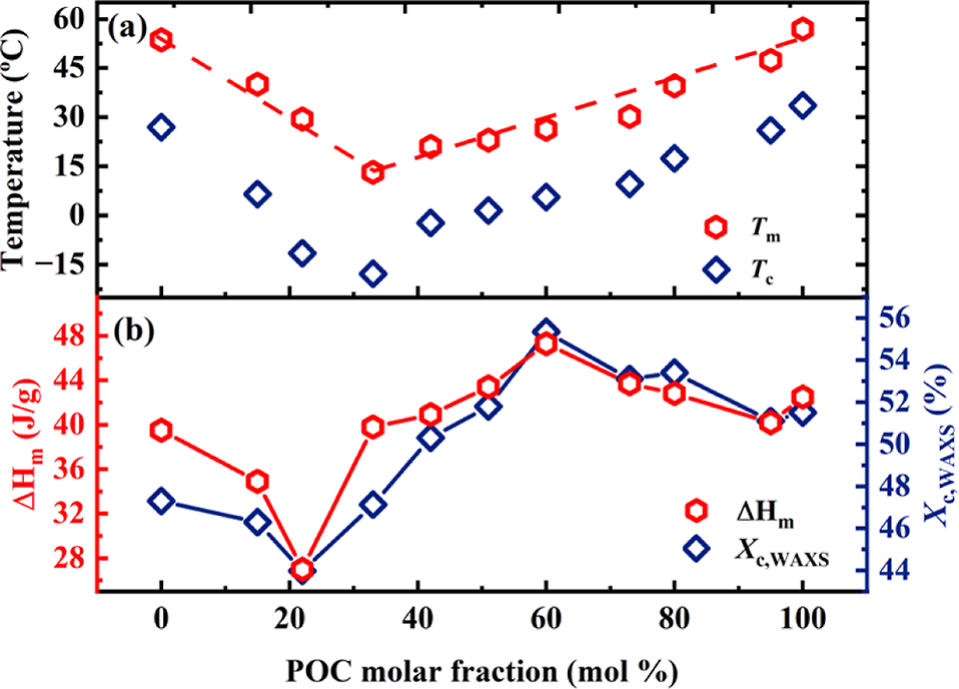
(a) *T*_c_ and *T*_m_ and (b) Δ*H*_m_ and *X*_c,WAXS_ as a function of POC composition. Lines have been drawn joining the data points to guide the eye.

The thermal transition temperatures extracted from Figure 2 are plotted as a function of the POC content in Figure 3. Figure 3a illustrates the correlation between the molar fraction of POC, i.e., the comonomer content, and the corresponding *T*_c_ and *T*_m_ of all materials. From a thermodynamic point of view, a pseudoeutectic-like behavior of the isodimorphic copolymers was observed, as characterized by a decreasing trend in *T*_c_ and *T*_m_ as the composition moves away from the two homopolymers of PHC and POC. This decreasing trend, which is also roughly observed in the composition-dependent behavior of the nucleation density on polarized light optical microscopy (PLOM) images (see Figure S5), is provoked by the changes in composition and the exclusion/inclusion competition. Minimum *T*_c_ and *T*_m_ values are observed for PH_67_O_33_C (with 33 mol % POC), considered the pseudoeutectic point. Such a low POC content in the eutectic copolymer suggests that the OC units, with their eight repetitive methylene units, play a dominant role compared to HC, with only six repeating methylene units, in the crystallization of the copolymers. Despite the POC dominance on copolymer crystallization, among copolycarbonates, the apparent pseudoeutectic position found in this work is higher than in other copolycarbonates, with a higher difference of methylene units between the parent components. But it is comparable to the 21–36 mol % of PC10 content found in PC8-*ran*-PC10 copolymers, also characterized by a difference of two methylene units between the parent components. One factor that can influence this behavior is the relative chain lengths of the two comonomers, as shown in previous studies on aliphatic copolyesters,^15,27,36^ and also the parity of the chain lengths of the two comonomers as has been recently highlighted in the literature.^19^ However, the factors that influence the position of the pseudoeutectic point still need to be completely understood, and their understanding is beyond the scope of this contribution. But further understanding of this issue will be presented in a future contribution, in which a diverse series of copolycarbonates synthesized using different diols with varying even and odd chain lengths will be studied.

Interestingly, as shown in Figures 2b and 3a, double melting peaks are not detected at the pseudoeutectic position, as often reported for other isodimorphic random copolymers. In PC7-*ran*-PC12 copolycarbonates, a similar observation from nonisothermal DSC corresponded to a coincident crystallization and melting, while WAXS detected two crystalline structures.^15^ More details regarding this behavior are discussed below.

Figure 3b shows an irregular Δ*H*_m_ variation (i.e., the copolymers display Δ*H*_m_ higher than the parent components and even show a maximum of around 60 mol % POC) with the composition, which does not correspond to the pseudoeutectic trend of the temperature vs POC content observed in Figure 3a. The equilibrium melting enthalpies (Δ*H*_m_°) of the copolymers needed to calculate the crystallinity degree by DSC (*X*_c,DSC_) have not yet been estimated yet. Still, they can be estimated through the Van Krevelen contribution group theory,^37^ as shown in Section S2. Despite the fact that *X*_c,DSC_ values might not include the comonomer inclusion/exclusion balance, they show the same trend as crystallinities values determined by WAXS (*X*_c,WAXS_) (see Figure S6). In this work, we considered the *X*_c,WAXS_ estimation to be more accurate. Therefore, the *X*_c,WAXS_ were calculated by fitting the peaks from the WAXS curves at −40 °C after cooling from the isotropic melt at 10 °C/min. Figure S7 gives an example of how the *X*_c,WAXS_ were obtained. As expected, in Figure 3b, the *X*_c,WAXS_ exhibits a similar variation trend as the Δ*H*_m_ with the increase of POC content. Compared to the PHC and POC homopolymers on both sides, *X*_c,WAXS_ initially decreases to two minimum values, corresponding to the PHC-rich copolymer PH_78_O_22_C and POC-rich copolymer PH_5_O_95_C. This can be attributed to the fact that some comonomer units act as defects excluded from the crystalline regions, limiting crystallinity and reducing the lamellar size. According to the literature, the crystallinity of isodimorphic copolymers also follows a pseudoeutectic behavior.^24^ Surprisingly, as the content of comonomers increases, the *X*_c,WAXS_ unexpectedly shows an upward trend instead of the expected downward one, reaching a maximum value for copolymer PH_40_O_60_C. This behavior is highly counterintuitive as the maximum *X*_c,WAXS_ value greatly surpasses that of both PHC and POC homopolymers despite the continued decrease in *T*_c_ and *T*_m_ toward the pseudoeutectic point. As shown below, this unique behavior is related to the presence of the third novel phase detected by WAXS and that shows a distinct conformation detected by FTIR.

### 3.3 Small-Angle X-ray Scattering and Wide-Angle X-ray Scattering

In situ WAXS/SAXS measurements were conducted to explore the crystalline structure using the same thermal conditions as those of the DSC test to ensure consistency.

**Figure 4:**
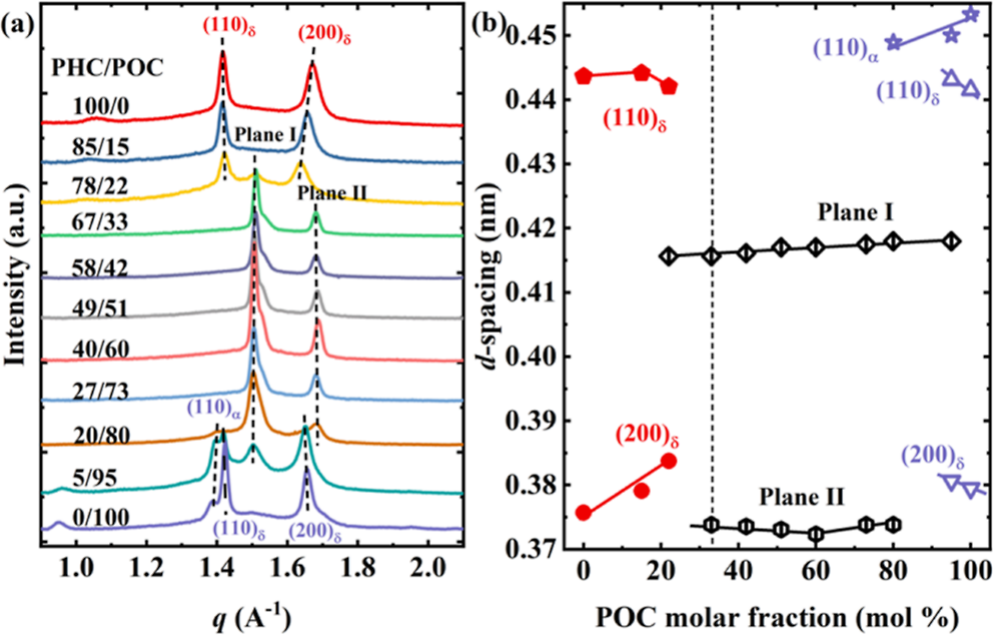
(a) WAXS profiles of all samples taken at 0 °C during the heating process after a previous cooling to −40 °C at 10 °C/min from the isotropic melt. (b) *d*-spacing of the indicated planes in (a) as a function of POC compositions. To differentiate between the crystal forms and planes of the two homopolymers, PHC and POC, which exhibit some similarities, two distinct colors were used: red for PHC and purple for POC. For several PH_*x*_O_*y*_C copolymers, a novel crystal form that is distinct from the two homopolymers was observed and denoted “Plane I″ and “Plane II” due to its unknown crystal structure and unit cell parameters.

Figure 4a compares the WAXS profiles of all materials obtained at 0 °C during heating. At this temperature, according to Figure 2b and the observations of Pérez-Camargo et al.,^32,33^ the diffraction pattern for both PHC and POC corresponds to the (110) and (200) planes of the δ-phase. According to their results,^32,33^ the δ-PHC/POC maintain similar crystalline forms as those of α-PHC and α-POC, but with more efficient chain packing, although the specific crystal structure of δ phase has not been reported. As an additional avenue of investigation, we aspire to elucidate their structures by conducting fiber diffraction using high-molecular-weight samples or by cultivating single crystals for electron diffraction; however, such a determination is outside the scope of the present work. Still, provisionally, α-PHC has been previously indexed to a monoclinic unit cell (*a* = 0.746 nm, *b* = 0.631 nm) and α = 60° with (110) and (200) as its main planes (*d*(110) = 0.441 nm and *d*(200) = 0.373 nm) due to the similarity between PHC and the known structure of the poly (hexamethylene carbonate-glycol) analogue.^38^

The crystalline structure of POC was determined by Zhao et al.^10^ They found that both α- and β-forms are monoclinic [α-form: *a* = 0.77 nm, *b* = 1.01 nm, *c* (fiber axis) = 2.52 nm and α = 31.5° with (110) and (200) as its main planes *d*(110) = 0.430 nm and *d*(200) = 0.380 nm; β-form: *a* = 0.81 nm, *b* = 0.89 nm, *c* (fiber axis) = 2.42 nm, and α = 31.9° with a single reflection from the (110/200) planes *d*(110/200) = 0.41 nm]. Figure 4a shows that a small amount of the α phase remains for POC, as indicated by the diffraction peak at *q* = 1.39 Å^–1^. A similar result was reported by Hung et al.^27^ and attributed to an incomplete α-δ solid–solid phase transition during the previous cooling process rather than the occurrence of reversible δ-α transition during heating.

Figure 4a shows that in the PHC-rich compositions, only the PH_85_O_15_C sample displays an analogous WAXS pattern to the PHC homopolymer, with two primary crystalline reflections at *q* = 1.42 and 1.67 Å^–1^ assigned to the (110) and (200) planes of the δ-phase. For the PH_78_O_22_C copolymer, even though the crystalline structure is dominated by PHC-type crystals, as evidenced by its two analogous main diffraction peaks, a newly observed peak at approximately *q* = 1.51 Å^–1^ suggests the presence of a new crystal phase. This signal does not correspond to the distinct signals from the PHC-type crystals.

A similar behavior as that observed for PH_78_O_22_C was reported in poly (decamethylene succinate-*ran*-decamethylene fumarate) copolymers.^28^ In this case, for DF contents of 37 and 57 mol %, the WAXS pattern displays a mixture of the main PDS and PDF reflections and extra reflections (like in this work) that do not belong to any of the parent components. Still, the authors claim that the copolymers were at pseudoeutectic conditions in these compositions rather than adopting a completely new crystalline form. In addition, slight changes in *T*_c_ and *T*_m_ were detected, corroborating that these copolymers seem to be at a wide pseudoeutectic region. Moreover, the melting enthalpy Δ*H*_m_ vs DF content displays a typical pseudoeutectic behavior. Thus, similarly to the literature, we can consider that the PH_78_O_22_C crystallizes in PHC-type crystals despite the extra peak. The extra peak might indicate the coexistence between PHC-type crystals and the new phase ones, as it occurs in the pseudoeutectic region or point of isodimorphic copolymers.

A unique and novel behavior is observed as the POC content increased between 33 and 73 mol %. For these compositions, only two prominent diffraction peaks at *q* = 1.51 and 1.68 Å^–1^ were detected; we denote them “Plane I″ and “Plane II” (see Figure 4a). These reflections do not correspond to any known crystalline diffraction planes of either PHC or POC or a mixture of them, as typically reported in isodimorphic copolymers. In addition, Planes I and II are also the main planes for the PH_20_O_80_C copolymer. Due to the lack of sufficient diffraction peaks, we cannot precisely determine this phase’s crystalline structure at present. However, by combining the results of FTIR, we propose a plausible hypothesis (see Section 3.4). Yet, until substantial experimental evidence is obtained, we continue to temporarily refer to this crystalline structure formed in these copolymers as the “new phase”. The WAXS results suggest the formation of a dominant new phase, distinct to PHC- and POC-type crystals, for an extensive range of compositions, i.e., POC contents of 33 to 80 mol %. The formation of this new phase coincides with the compositions in which the *X*_c,WAXS_ vs POC content (see Figure 3b) behavior deviates from the expected one (e.g., a pseudoeutectic behavior), exhibiting an “inverse” pseudoeutectic trend. Interestingly, despite forming a new phase for such an extensive composition range, these copolymers continue displaying a pseudoeutectic behavior in temperatures vs POC content plots (see Figure 3a).

For the POC-richest compositions, both copolymers, PH_5_O_95_C and PH_20_O_80_C, generate a mixture of POC-type crystals and a new crystal phase during crystallization. But the dominant crystalline phase is different.

The PH_20_O_80_C copolymer, which crystallizes in the new phase-dominant crystals, also displays a small diffraction peak at *q* = 1.41 Å^–1^, indicating the coexistence of the POC-type crystals. In contrast, the PH_5_O_95_C copolymer crystallization is dominated by POC-type crystals. It exhibits the characteristic peak of the new crystal phase at *q* = 1.51 Å^–1^, together with the diffraction peaks of the δ and α phases of homopolymer POC. Specifically, the peaks at *q* = 1.39 Å^–1^ belong to the (110) plane of the α phase, whereas the diffraction peaks at *q* = 1.42 and 1.65 Å^–1^ were attributed to the (110) and (200) planes of POC δ phase, respectively. Notably, in the homopolymer of POC, the induction of the new phase crystallization requires only 5 mol % of HC units, while in the case of PHC, a critical concentration of 22 mol % OC units is required to induce the formation of new phases.

Figure 4b shows the interplanar distances (of the planes indicated in Figure 4a), *d*-spacings, calculated with Bragg’s law plotted as a function of the POC content. For clarity, we separately analyzed the three regions found in Figure 4a: PHC-rich, POC-rich, and new-phase regions.

For PHC-rich copolymers, *d*(200)_δ_ increases as the POC content increases, whereas *d*(110)_δ_ slightly increases and then decreases. This enlargement in the *d*-spacing values indicates that PHC crystal unit cells must expand their volume (i.e., *a* and *b* sizes) to incorporate larger OC units. Analogous *d*-spacings increase with composition to accommodate comonomer inclusion has also been reported for other random copolymers.^15,19,23,27^ In the case of POC-rich compositions, *d*-spacing values (*d*(110)_δ_ and *d*(200)_δ_) also increase with PHC content due to the inclusion of PHC counits. The changes in *d*(110)_α_ values as PHC content increases also are provoked by PHC counits incorporation; otherwise (i.e., comonomer exclusion), the *d*-spacing should remain unchanged.

Within the range of intermediate compositions (22–95 mol % POC), where PH_*x*_O_*y*_Cs can crystallize into the new phase, an upward trend is observed in the spacing of Plane I as POC compositions increase, resembling the findings in isomorphic systems.^19,20^ In contrast, the *d*-spacing values of Plane II initially decrease to a minimum at PH_40_O_60_C before subsequently increasing.

**Figure 5:**
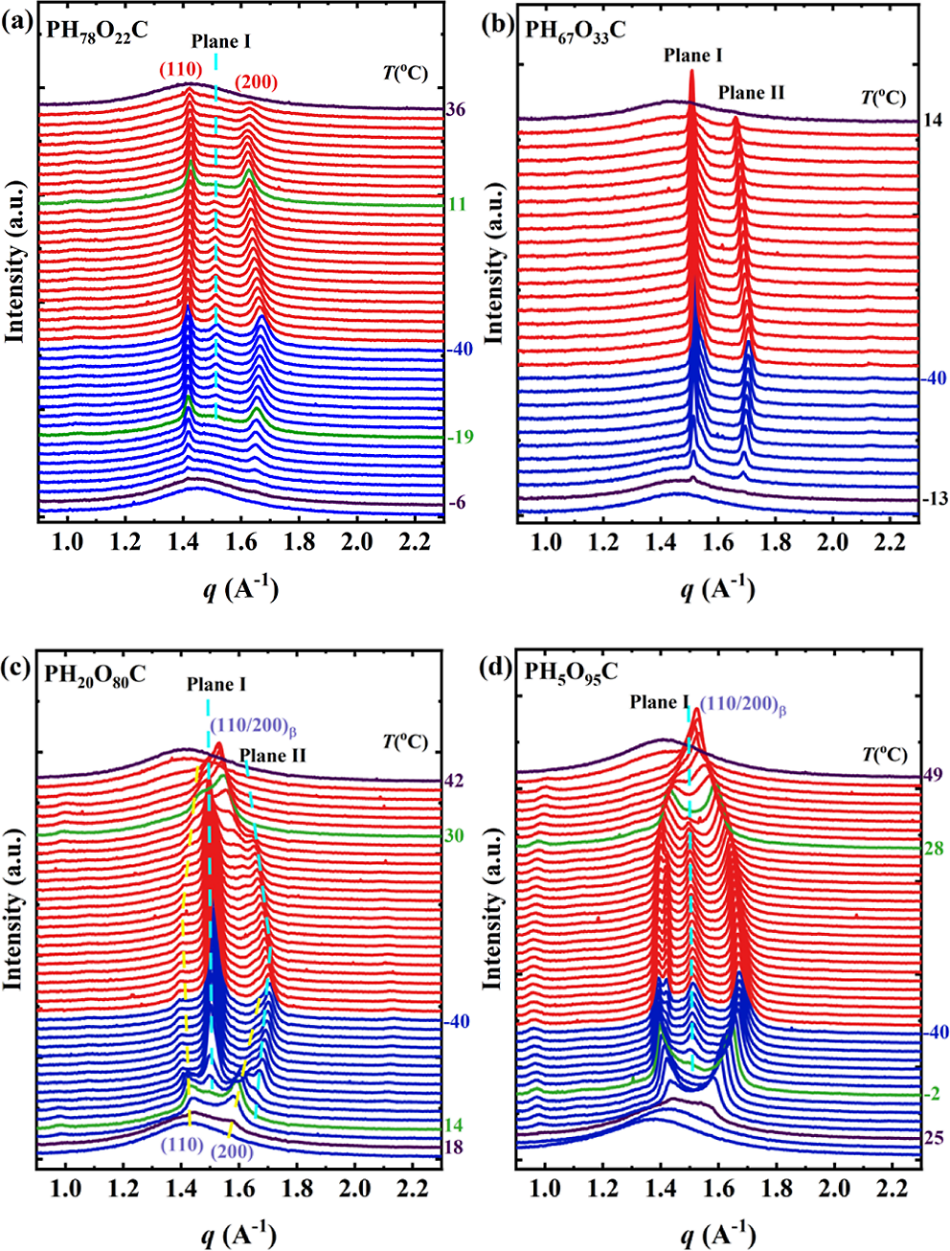
Temperature-dependent WAXS diffractograms acquired during cooling and heating at 10 °C/min for (a) PH_78_O_22_C, (b) PH_67_O_33_C, (c) PH_20_O_80_C, and (d) PH_5_O_95_C. The blue curves represent the cooling process, while the red curves represent the subsequent heating process. The two green curves in both the cooling and heating processes represent the critical temperatures for the appearance and disappearance of the new crystal phase, respectively. The black curves indicate the initial temperature of crystallization (*T*_c,initial_) and the final melting temperature (*T*_m,final_), defined as the first temperature at which a crystalline signal appeared and the first temperature at which the crystalline signal disappeared, respectively. This notation is employed to differentiate between WAXS and DSC experiments. The corresponding temperature values are also marked on the side.

In situ temperature-dependent WAXS was performed to understand further the formation of the new crystalline phase and the relationship with the PHC and POC solid–solid transitions. Figure 5 shows the WAXS patterns collected during cooling and heating, at 10 °C/min, for representative samples. The temperature-dependent WAXS profiles of the remaining samples are presented in the Supporting Information (Figure S8).

Figure 5a illustrates that during cooling of the PH_78_O_22_C copolymer, two diffraction peaks assigned to PHC-type crystals appear starting from −6 °C, while the appearance of a diffraction peak at *q* = 1.51 Å^–1^ from −19 °C indicates the formation of the new phase. Next, this new phase and the PHC-type crystals subsequently melted at 11 and 32 °C, respectively, while no thermal signal was observed at 11 °C in the DSC heating scan. Moreover, the FTIR variable-temperature spectra shown in Figure S12a during the heating from 10 to 15 °C did not reveal any changes in the chain packing mode. Therefore, the imperceptible thermal signal might be attributed to the small amount of the new phase, causing its melting peak to be overlapped by the broad thermal transition of melting and recrystallization that initiates at around 5 °C. The small endothermic peak at 23.2 °C does not fit the vanishing temperature of the diffraction peak, as shown in Figure 2b and Table 2. These results support the assertion that the double melting peaks arise from melting recrystallization rather than polymorphism.

Initial examination of the diffraction pattern collected at 0 °C for the PH_67_O_33_C copolymer, as depicted in Figure 4, demonstrates its crystallization as a pure new crystalline phase. Of particular note is the absence of characteristic peaks associated with known PHC- or POC-type crystals throughout the applied thermal treatment shown in Figure 5b. This observation effectively precludes the possibility of the new crystal phase being derived from the phase transition of the known crystal structures. The evidence presented in the in situ results suggests that the new phase in PH_67_O_33_C was directly generated via melt crystallization. In addition, the characteristic single strong peak corresponding to the β-POC phase (see Figure S8g) is absent, indicating that such a solid–solid transition does not occur in these materials. An analogous behavior is observed for PH_58_O_42_C, PH_49_O_51_C, PH_40_O_60_C, and PH_27_O_73_C (see Figure S8c,f).

Figure 5c shows that PH_20_O_80_C copolymer crystallization starts with forming POC-type crystals as indicated by the signal of the (110) and (200) planes at 18 °C. But at 14 °C, the new crystalline phase starts forming (see Planes I and II). As the temperature decreases, the coexistence of the POC-type and the new phase-type crystals occurs, with dominance of the new phase-type crystals. Upon heating, the new phase-type crystals melt before (∼30 °C) the POC-type ones (∼41.8 °C). At around 28 °C, an additional indicator of the dominance of the remaining POC-type crystals is the strong peak [(110/200)_β_] corresponding to the β-POC phase crystals, i.e., Brill-like transition. The observed melting temperatures in the in situ WAXS experiments are consistent with the endothermic peaks detected during the second heating DSC scans (see PH_20_O_80_C in Figure 2b and *T*_m,end_ values in Table 2). This observation demonstrates that the smaller melting peak detected during the second heating DSC scan can be attributed to melting of the new phase.

A different situation is observed for PH_5_O_95_C since the POC-type crystals dominate the crystallization. In this case, POC crystals are formed at ∼25 °C (see peaks generated by (110) and (200) planes in Figure 5d), and at a lower temperature, ∼ −2 °C, a peak from the new phase (see the peak generated by Plane I in Figure 5d) is detected. Upon heating, the peak of the new phase melts at 28 °C, followed by the melt of POC-type crystals at 49 °C. This latter value is in line with DSC results (see Figure 2b). Figure 5d also shows the characteristic signal generated by the β-POC phase [see the peak generated by the (110/200)_β_ plane], confirming the dominance of POC-type crystals.

**Figure 6:**
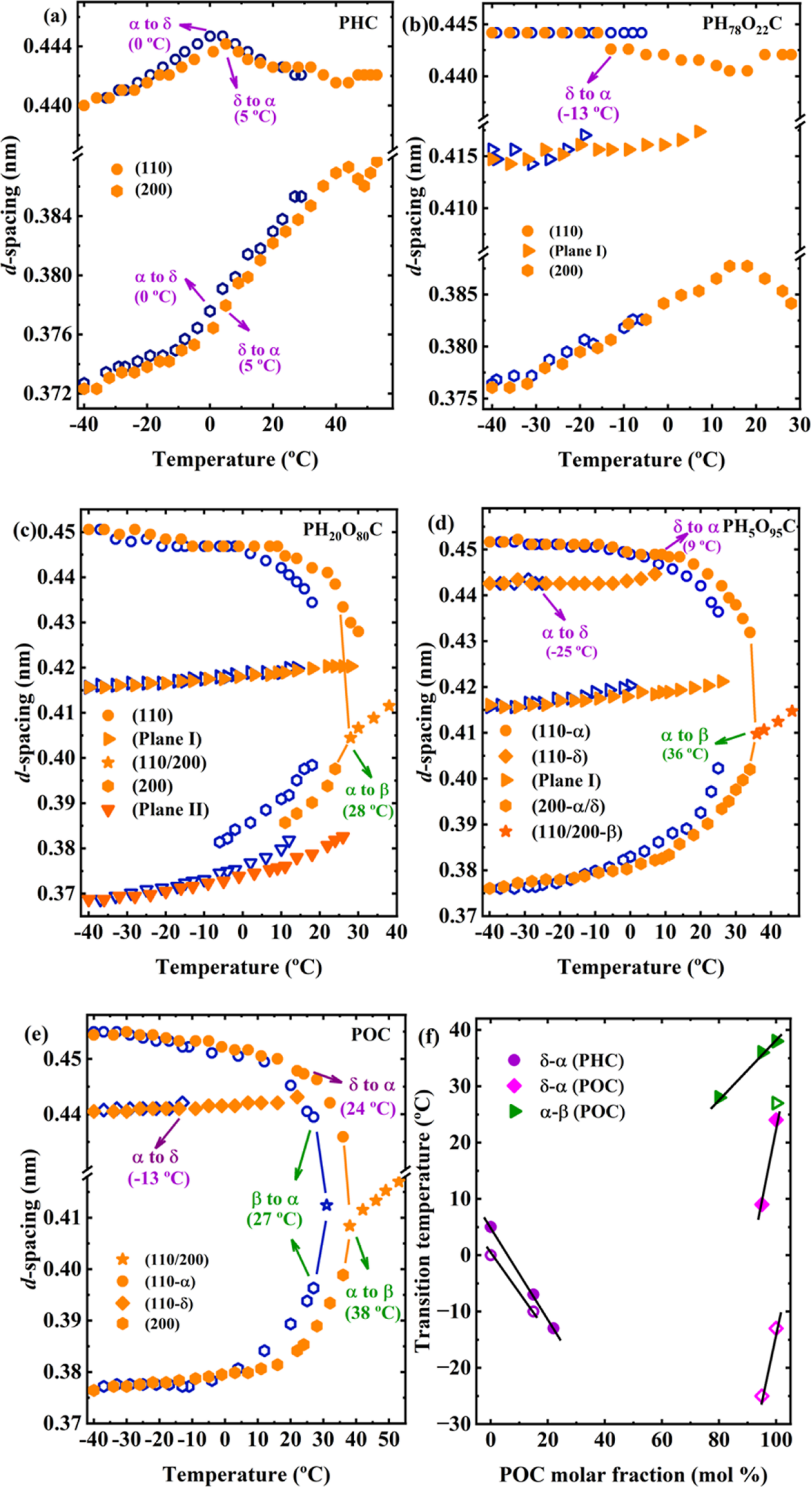
*d*-Spacing evolution for (a) PHC, (b) PH_78_O_22_C, (c) PH_20_O_80_C, (d) PH_5_O_95_C, and (e) POC. The blue open points represent the cooling process, while the orange solid points correspond to the heating process. (f) Solid–solid phase transition temperatures as a function of POC molar fraction. In (f), the solid points indicate the phase transition occurring during the cooling process (β-α and α-δ transitions), while the open points represent the corresponding reversible transition process during heating (δ-α and α-β transitions).

In Figures 5 and S8, the α to β solid–solid transition of the POC is easily observable. However, PHC and POC also possess a δ to α transition, which is clear in *d*-spacing vs temperature plots. Figure 6 presents the evolution of *d*-spacing for each plane in representative copolymers of PH_78_O_22_C, PH_20_O_80_C, and PH_5_O_95_C as well as two parent homopolymers of PHC and POC to clarify the influence of comonomer inclusion on the phase transition. Interestingly, as shown in Figure S9, none of the copolymers that crystallize with only the new phase crystals undergo δ to α or α to β transitions, which is further evidence of the absence of PHC or POC features.

Figure 6a shows that for the PHC homopolymer, the *d*-spacing variation of the (110) plane at around 0 °C indicates the occurrence of α to δ phase (cooling) transition, while the reversible δ to α phase (heating) transition was observed at 5 °C. The transition temperatures are also roughly consistent with the marked peaks in Figure 2. The δ to α transition is not clear for PH_78_O_22_C (see Figure 6b) since *d*(110) remains unchanged during cooling, except for a sudden decrease in the *d*(110) at −13 °C, which might imply the occurrence of the δ to α transition.

Regarding the POC homopolymer, Figure 6e clearly illustrates the multiple reversible solid phase transition, which follows the β-α-δ sequence during the cooling process. Interestingly, with the incorporation of the HC counit, the β-α phase transition process was not observed during the cooling process for PH_20_O_80_C and PH_5_O_95_C copolymers despite the occurrence of the anticipated α-β phase transition during the subsequent heating process. At the onset of crystallization, i.e., *T*_c,initial_, the first WAXS profile in Figure 5c,d displays two diffraction peaks at approximately *q* = 1.44 and 1.58 Å^–1^, demonstrating the absence of the β phase. The POC homopolymer and PH_5_O_95_C copolymer both demonstrated a reversible α-δ phase transition, whereas the occurrence of this phase transition in PH_20_O_80_C remains unclear due to the absence of discernible changes in the *d*-spacing of (110) and (200) planes.

Even though complementary FT-IR (here focused on the chain conformation; see Section 3.4) studies are needed to understand the solid–solid transition mechanism in the copolymers, analyzing, structurally, solid–solid transition temperature evolution with comonomer content can be useful to gain some insights into their behavior. Figure 6f shows the temperatures for reversible δ-α and α-β phase (*T*_δ-α_ and *T*_α-β_) transitions (indicated in Figure 6a–e) as a function of POC content. The gradual incorporation of HC counits leads to a reduction in both *T*_δ-α_ and *T*_α-β_. Similar behavior is observed for the *T*_δ-α_ when OC counits are incorporated into a PHC-rich copolymer. Considering the findings of Peréz-Camargo et al.,^32,33^ it can be inferred that, by analogy, a decrease in *T*_δ-α_ with crystal perfection can be related to less perfect crystals as a result of the comonomer inclusion. Interestingly, in PC8-*ran*-PC10 copolymers, Hung et al.^27^ found an increase in the *T*_α-δ_ as C10 content increased, while no significant changes were found in *T*_α-β_ after isothermally crystallizing the samples at 35 °C followed by cooling (to −50 °C) and heating (to 63 °C) at 2 °C/min. This behavior was attributed to a disordered chain conformation (incorporating more gauche chain conformations) when the C10 content is increased. However, the thermal history influence was not considered.

These interesting results demonstrated that the variation of the solid–solid transition in copolymers depends on the comonomer inclusion/exclusion balance. Probably, C10 counits have more difficulties entering POC-type crystals than the smaller PHC counits; thus, the solid–solid transitions are affected differently.

**Figure 7:**
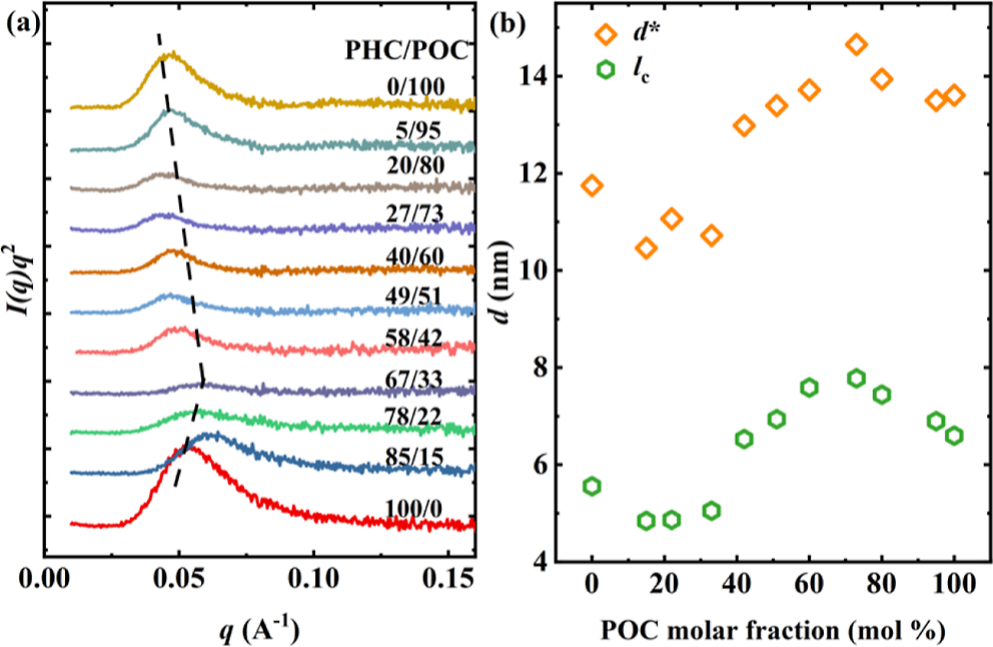
SAXS patterns of all samples recorded at −40 °C cooling from the isotropic melt at 10 °C/min: (a) SAXS patterns; (b) long periods, d*, and lamellar thickness, *l*_c_, as a function of POC compositions. The dashed lines are drawn to guide the eyes.

We further investigated the lamellar structures of all samples. SAXS patterns collected at −40 °C, after the sample was cooled from the isotropic melt at 10 °C/min, are shown in Figure 7a. In all cases, only one scattering peak (*q*) was observed, arising from the X-ray scattering of the lamellar stacks. In the case of PH_78_O_22_C, PH_20_O_80_C, and PH_5_O_95_C copolymers, where two crystalline forms can crystallize at −40 °C, SAXS shows only one scattering peak, as in other systems,^22^ since only an average long period can be detected. A single SAXS maximum was also observed during in situ SAXS measurements (see Figure S10) taken during the cooling and heating of the samples.

The position of the SAXS maximum changes with composition, indicating that the comonomers provoke changes over a long period. The long period (d*) values were estimated from the position of the scattering peak in the Lorentz-corrected SAXS patterns (*I*(*q*)*q*^2^ vs *q*), according to eq 6.
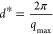
6

Using eq 7, the lamellar thickness *l*_c_ has also been calculated and plotted in Figure 7b
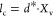
7where *X*_v_ is the crystalline volume fraction, which we have approximated to the mass fraction of crystals (*X*_c,WAXS_). Figure 7b shows the evolution of d* and lamellar thickness, *l*_c_, as a function of POC content, demonstrating their dependence on composition. The found trends are similar to those observed in *X*_c,WAXS_ vs POC content, reflecting that those compositions that crystallize with a new crystalline phase possess higher *l*_c_.

As mentioned above, the *l*_c_ values for those copolymers that form a new crystalline phase are higher than those of the parent components. This behavior differs from the expected for isodimorphic copolymers. Moreover, it differs from the *T*_m_ vs POC content behavior.

### 3.4 Fourier Transform Infrared Spectroscopy

Figure S11 shows the spectra recorded, in the 4000 to 650 cm^–1^ range, at −40 °C, for four representative copolymers: PH_78_O_22_C (with a mixture of PHC-type and new phase), PH_67_O_33_C (pure new phase at the eutectic point), PH_49_O_51_C (pure new phase at the middle composition), and PH_20_O_80_C (mixture of POC-type and new phase). The absorption bands at around 2857 and 2933 cm^–1^ are associated with the symmetric and asymmetric –CH_2_– stretching vibrations of methylene groups, respectively.^4^ In the case of the carbonate groups, the C=O stretching and O–C–O asymmetric stretching vibrations are observed within the ranges of 1770 to 1710 and 1317 to 1211 cm^–1^, respectively.^4,39^

**Figure 8:**
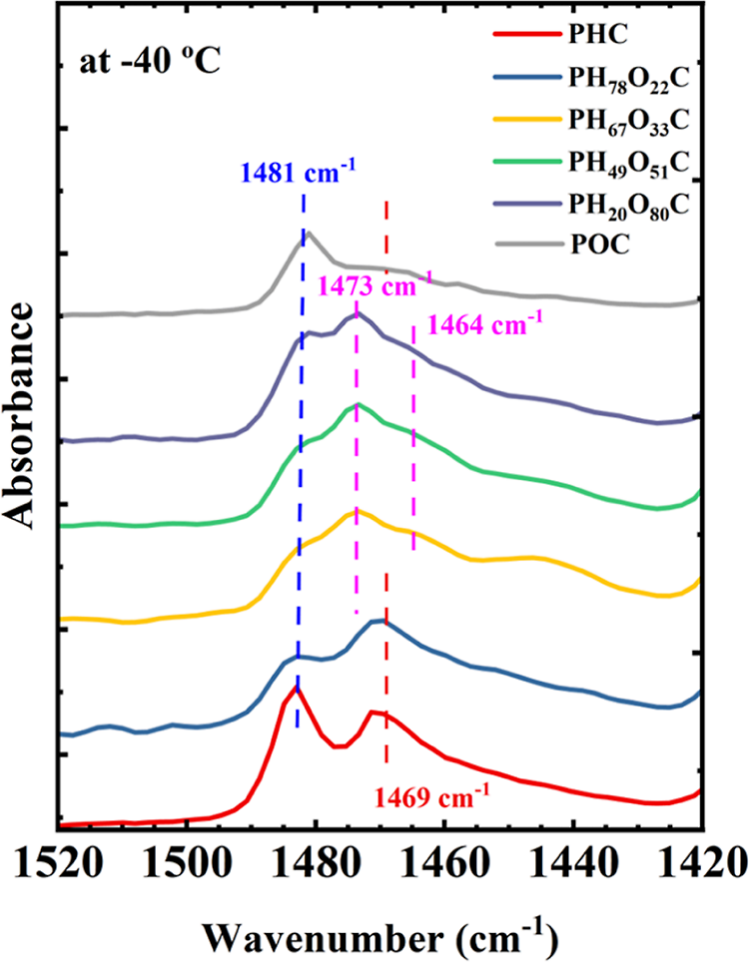
FTIR spectra of selected copolymers PH_78_O_22_C, PH_67_O_33_C, PH_49_O_51_C, and PH_20_O_80_C along with the parent homopolymers of PHC and POC in the range 1500–1300 cm^–1^. The spectra were recorded at −40 °C.

This work focused on the changes in the methylene group’s conformation, which are distinguished in the CH_2_–bending region by the absorption bands associated with *trans* or *gauche* conformers.^13^Figure 8 shows the FTIR spectra of the typical copolymers and the parent homopolymers of PHC and POC (data extracted from our previous paper^13,32^), covering the range of 1500–1300 cm^–1^. Variable-temperature FTIR spectra are given in Figure S12. The absorption bands at 1481 and 1466 cm^–1^ in PHC and POC can be assigned to the *trans* and *gauche* conformations of the methylene group, respectively, as reported previously.^13^ The relatively stronger bands at 1481 cm^–1^ for PHC and POC suggest that the *trans* conformation is dominant, agreeing with the WAXS results and previous studies on the δ phase at −40 °C. Both homopolymers also show a *trans* conformation dominance, although weaker, in the α phase.

Interestingly, for those copolymers that crystallize in the new phase (PH_67_O_33_C and PH_49_O_51_C copolymers), two absorption bands at around 1473 and 1463 cm^–1^, which are absent in the homopolymers PHC and POC, are noticeable, and they are strong, whereas the 1481 cm^–1^ band becomes weaker. The temperature-dependent experiments (Figure S12b,c) show that these two new bands appear at the beginning of the crystallization, indicating that they are crystalline bands related to the ordered chain packing of the new phase. This observation is consistent with the findings revealed by the WAXS patterns in Figures 4a, 5b, and S8, indicating the formation of a new crystal phase in those samples with intermediate composition. These two bands are very similar to those exhibited by polyethylene (PE) crystals and stem from a factor group splitting due to the packaging of two polymer chains in an orthorhombic lattice.^40,41^ Additionally, the diffraction peaks at *q* = 1.51 and 1.68 Å^–1^ and their intensity bear a striking resemblance to orthorhombic PE.^42^ Consequently, we cautiously speculate that the new phase forming in these copolymers might involve molecular chains adopting a PE-like conformation, packed in a lattice resembling orthorhombic PE crystals.

In the case of PH_20_O_80_C, in addition to the 1473 cm^–1^ band, the band at 1481 cm^–1^ is stronger than those in PH_67_O_33_C and PH_49_O_51_C. This indicates the coexistence of the POC-type crystal and the new phase, as evidenced by the WAXS results in Figures 4a and 5c. In contrast, for PH_78_O_22_C, the characteristic bands from the new phase were not identified, which could be attributed to a small amount of the new phase in the sample, as supported by the notably faint diffraction peak at *q* = 1.51 Å^–1^ in Figure 5a. Instead, Figure 8 shows for PH_78_O_22_C that the band at 1469 cm^–1^ is more pronounced, suggesting a *gauche* conformation dominance provoked by the mixture of the PHC-type and new phase-type conformation. A more profound analysis of these conformational changes and the solid–solid transition evolution transgresses from the aim of this paper, and it will be presented in a future contribution.

### 3.5 Understanding the Crystallization Behavior of the New Crystalline Phase

The crystallization behavior of PH_*x*_O_*y*_C copolymers challenges the concept of isodimorphism. On the one hand, the thermal characterization, specifically in thermal transitions vs POC content plots, displayed the typical pseudoeutectic behavior, with crystallization in all the composition ranges and a pseudoeutectic point at 33 mol % POC content. On the other hand, a first estimation of the crystallinity shows a unique behavior since a wide range of compositions (33 to 73 mol % of POC content), including the pseudoeutectic point, possesses higher crystallinity values than the parent components. Moreover, the in situ WAXS characterization revealed that for all the above compositions (33 to 73 mol % of POC), a new crystalline phase has been formed, completely different from the parent components’ crystal structure.

The behavior described above is unexpected. In isodimorphic copolymers, the crystallinity normally shows a pseudoeutectic dependence with comonomer content as the exclusion/inclusion balance is a function of composition. A mixture of A-type and B-type structures has only been reported at the pseudoeutectic point or region for isodimorphic random copolymers. These structures melt sequentially upon heating, even if such sequential melting is not reflected in *T*_m_ vs comonomer content as in PC7-*ran*-PC12 copolymers. Only in one case, the appearance of a new WAXS diffraction signal has been reported but together with the presence of the parent components’ reflections.^28^ This work reports a similar situation for PH_78_O_22_C, PH_20_O_80_C, and PH_5_O_95_C, showing a WAXS pattern dominated by PHC- or POC-type crystals with a trace of the new phase crystals and vice versa. Yet, the formation of a new crystalline phase totally different from those of the parent components has never been reported for isodimorphic copolymers, as far as the authors are aware.

**Figure 9:**
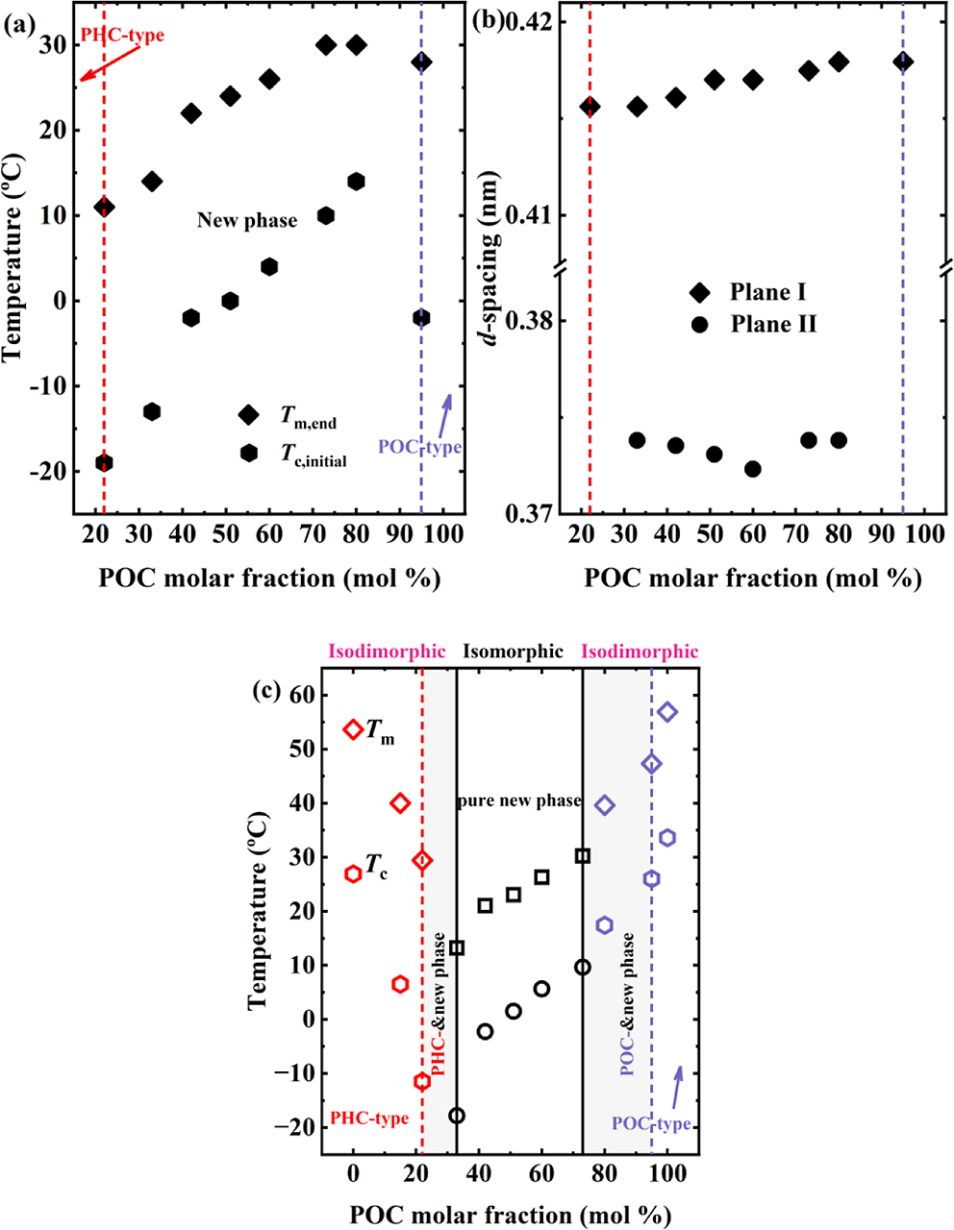
(a) *T*_c,initial_ and *T*_m,final_ of the new phase and (b) *d*-spacing of Plane I and Plane II of the new phase, in copolymers capable of crystallizing to this type of new phase as a function of POC composition (solid points extracted from in situ WAXS profiles). (c) *T*_c_ and *T*_m_ as a function of POC composition (open points extracted from the DSC traces). The critical compositions for forming pure PHC- or POC-type crystals are indicated by the red and blue dashed lines, respectively. In (c), the solid black lines indicate the boundary for forming the pure new phase, and the shaded areas represent the mixed phase, denoting the coexistence of PHC-type crystals and the new phase or POC-type crystals and the new phase.

On the basis of the random copolymer crystallization modes, the formation of a new crystalline phase is a typical feature of isomorphism. Another important feature of isomorphic copolymers is that the thermal properties and structural parameters increase linearly as the comonomer content increases. In this case, if we consider the wide range in which the new phase is formed, we can find the isomorphic characteristics displayed in Figure 9.

Figure 9a shows *T*_c,initial_ and *T*_m,final_ (detected by WAXS) vs POC content for those copolymers that can crystallize with the new crystalline phase. Similar to an isomorphic system, within the labeled composition range, *T*_c,initial_ and *T*_m,final_ increase as the POC content increases. Figure 9b plots the *d*-spacing vs POC content, exhibiting a similar trend, although the change is small. Therefore, the evidence suggests a high degree of comonomer inclusion, or even total cocrystallization, in the composition range where the new crystalline phase is found.

Considering all of the compositions, a hypothesis may explain the peculiar behavior of the PH_*x*_O_*y*_C copolymers. Phenomenologically, our system can be regarded as a combination or mixture of two crystallization modes: isodimorphism and isomorphism. As illustrated in Figure 9c, the PH_5_O_95_C crystallizes in a purely isodimorphic mode, decreasing its *T*_c_, *T*_m_, and *X*_c,WAXS_ due to PHC counits inclusion. The PH_20_O_80_C seems to be in a transition region between isodimorphism and isomorphism, as evidenced by the decreases in *T*_c_ and *T*_m_ but the increase (to higher values than the neat POC) in *X*_c,WAXS_. In addition, the PH_20_O_80_C crystal structure is dominated by the new crystalline phase type, but still, it shows the characteristic β-phase of the POC. At 33 mol % ≤ POC content ≤73 mol %, the copolymers crystallize in a purely new phase (without traces of POC or PHC). At POC content = 22 mol %, the PH_78_O_22_C copolymer crystallization is dominated by PHC-type crystals but still shows a crystalline peak characteristic of the new phase; hence, this point is the transition to an isodimorphic crystallization but dominated by the PHC-type crystals. The PH_85_O_15_C copolymer crystallizes in purely PHC-type crystals and an isodimorphic mode.

To fulfill the request for the current situation, thermodynamically, the crystals with total inclusion should be more stable than those with partial inclusion and maintain the parent crystalline structure. Although, as displayed in Figure S13, a linear relationship between *T*_m_ and comonomer content has been reported; so far, in isomorphic systems, the theoretical scenario of the equilibrium melting temperature of isomorphic copolymers has not been established. Especially, the total inclusion of comonomers in a crystal with random comonomer distribution may indicate nonidentical unit cells, similar to the case of a solid solution. Therefore, a nonlinear relation of *T*_m_ vs composition is not theoretically impossible.

Another factor is the composition of crystals, which can differ from the average composition of the polymers. Considering the small changes in the lattice spacing in the new phase, one may conjecture that the compositions of the crystals in those samples are comparable. If this is true, because the comonomer content varies from 22 to 80 mol %, the amount of chain sequences with the “right” comonomer content differs accordingly, which would result in varied crystallinity. A supportive clue can be found in Figure 3b, where *X*_c,WAXS_ increases with the POC molar fraction in the new phase region and reaches the maximum at 60 mol %. This maximum may correspond to the “right” composition for cocrystallization.

The above hypothesis needs further verification both theoretically and experimentally. Nevertheless, the new findings in this work manifest the complexity of the crystallization of random copolymers. It shows that when the penalty of including comonomers in a parent unit cell exceeds that of creating a new, maybe less ordered, crystalline phase, the system will follow a new crystallization pathway.

## 4 Conclusions

In this work, novel PH_*x*_O_*y*_C copolymers were synthesized in a wide range of compositions by a two-step melt polycondensation method. The copolymer compositions were determined by using ^1^H NMR spectra, and ^13^C NMR characterization confirmed that all copolymers have a random microstructure. The molecular weights and dispersities of the materials were measured using SEC, ensuring that they were comparable for analysis purposes.

The DSC results show that all samples can crystallize regardless of the compositions, which is typical of isodimorphic and isomorphic copolymers. The crystallization and melting temperatures as a function of POC content vary in a pseudoeutectic way, showing minimum values (pseudoeutectic point) in the PH_67_O_33_C copolymer (67 mol % PHC and 33 mol % POC). This behavior is characteristic of isodimorphic copolymers. However, interestingly, the crystallinity variation and structural characterization show novel and unique behavior. For a wide range of compositions (33 to 73 mol % of POC content), including the pseudoeutectic point, the *X*_c_ of the copolymers (estimated either by DSC or WAXS) is higher than the *X*_c_ of the parent components, instead of showing a pseudoeutectic behavior. For these copolymers, the WAXS analysis revealed a new crystalline form, with two main diffraction peaks at around *q* = 1.51 and 1.68 Å^–1^, different from those of PHC- and POC-type crystals. This new crystalline form does not show the characteristic solid–solid transition of the PHC- and POC-type crystals, e.g., in those copolymers with POC composition of 33–73 mol %, including the pseudoeutectic point. This suggests that a new crystalline phase distinct from those of two-parent homopolymers was found for those copolymers with intermediate compositions.

Temperature-dependent WAXS and FTIR spectroscopy confirmed the formation and melting of the new crystal phase. FTIR experiments revealed that those copolymers that crystallize with a new phase display a PE-like chain conformation, which differs from the homopolymers that adopt a *trans*-dominant conformation. Thus, considering WAXS and FTIR results, the new phase resembles an orthorhombic PE-type crystal. Interestingly, for those copolymers crystallized into pure new phases (33–73 mol % POC), the melting temperature linearly increases with the increase of POC concentration. The linear increase in the melting temperature of the new phase with the incorporation of the POC composition implies that local isomorphism emerges in an isodimorphic copolymer, which has never been observed in other systems. Thus, PH_*x*_O_*y*_C copolymers crystallize in a unique combination of isodimorphism and isomorphism modes.

## Supporting Information Available

^1^H NMR and ^13^C NMR spectra of samples, PLOM images, an example of peak fitting analysis of WAXS profiles, in situ WAXS/SAXS profiles and corresponding *d*-spacing evolution as a function of temperature, and variable-temperature FTIR spectra (PDF)
