# Supplementary material for: Challenging Isodimorphism Concepts: Formation of Three Crystalline Phases in Poly(hexamethylene-ran-octamethylene carbonate) Copolymers
Source: Macromolecules. 2023 Oct 11;56(20):8199–213. doi: 10.1021/acs.macromol.3c01265 (PMC10601535; doi:10.1021/acs.macromol.3c01265)
Supplement: Supplementary file 1 — ma3c01265_si_001.pdf [file ma3c01265_si_001.pdf]

Challenging Isodimorphism Concepts: Formation of three Crystalline Phases in Poly (hexamethylene-*ran*-octamethylene carbonate) Copolymers

Yilong Liao[1], Ricardo A. Pérez-Camargo[1], Haritz Sardon[1], Antxon Martínez de Ilarduya[2], Wenxian Hu[3,4], Guoming Liu[3,4], Dujin Wang[3,4], Alejandro J. Müller[1,5*]*

[1] POLYMAT and Department of Polymers and Advanced Materials: Physics, Chemistry, and Technology, Faculty of Chemistry, University of the Basque Country UPV/EHU, Paseo Manuel de Lardizábal, 3, 20018 Donostia-San Sebastián, Spain.

[2] Department of Chemical Engineering, Polytechnic University of Catalonia ETSEIB-UPC, Diagonal 647, 08028 Barcelona, Spain.

[3] Beijing National Laboratory for Molecular Sciences, CAS Key Laboratory of Engineering Plastics, Institute of Chemistry, Chinese Academy of Sciences, Beijing 100190, P. R. China.

[4] University of Chinese Academy of Sciences, Beijing 100049, P. R. China.

[5] Ikerbasque, Basque Foundation for Science, Plaza Euskadi 5, 48009 Bilbao, Spain.

*Corresponding authors: gmliu@iccas.ac.cn, alejandrojesus.muller@ehu.es

Section S1. Chemical Characterization of Copolymers

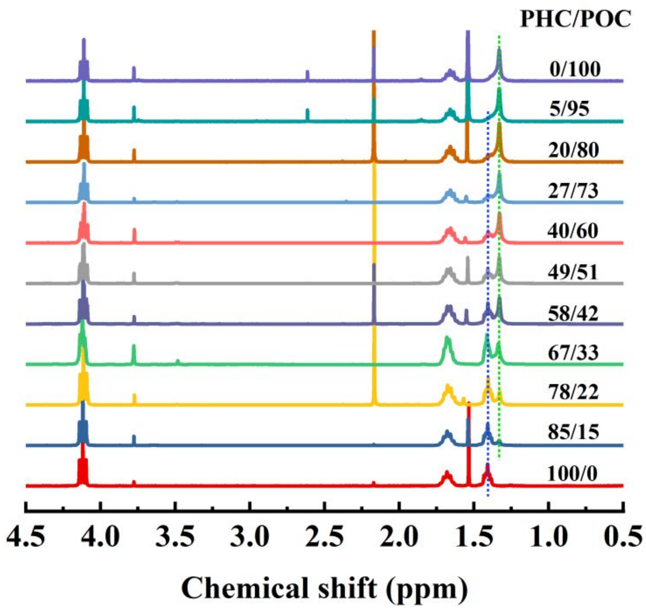

Figure S1. ^{1}H NMR spectra of all materials in $CDCl_3$.

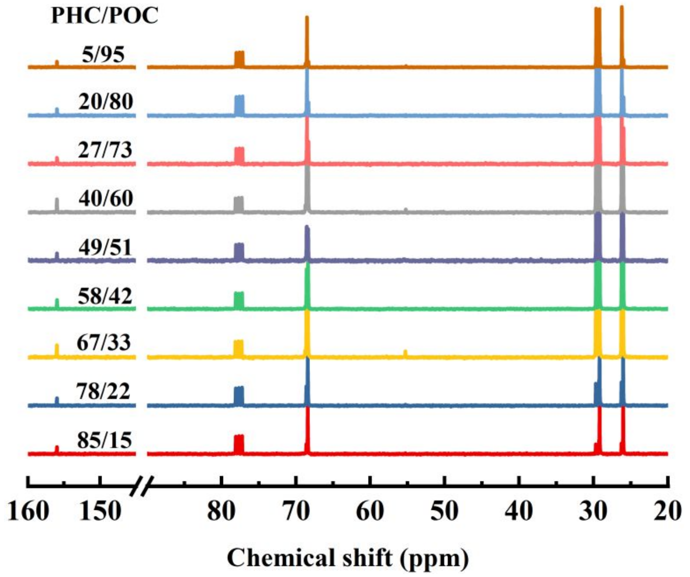

Figure S2. ^{13}C NMR spectra of all materials in $CDCl_3$.

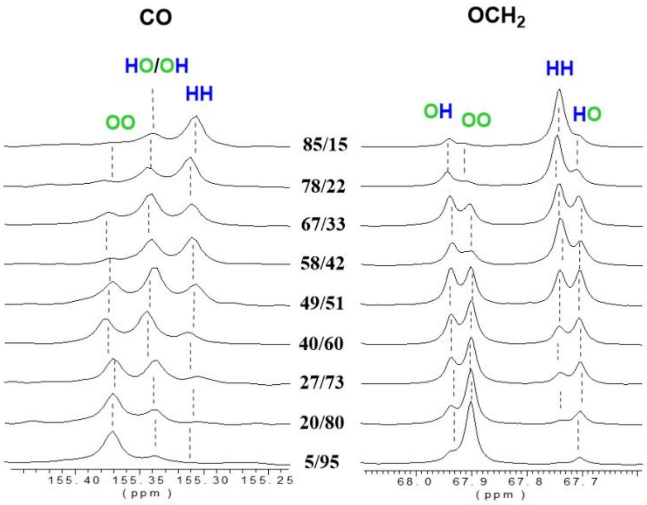

Figure S3. The assignments of carbonyl **<u>C</u>**O and –O**<u>C</u>**H_2 carbon resonance in ^{13}C NMR spectra for PH_xO_yC copolymers.

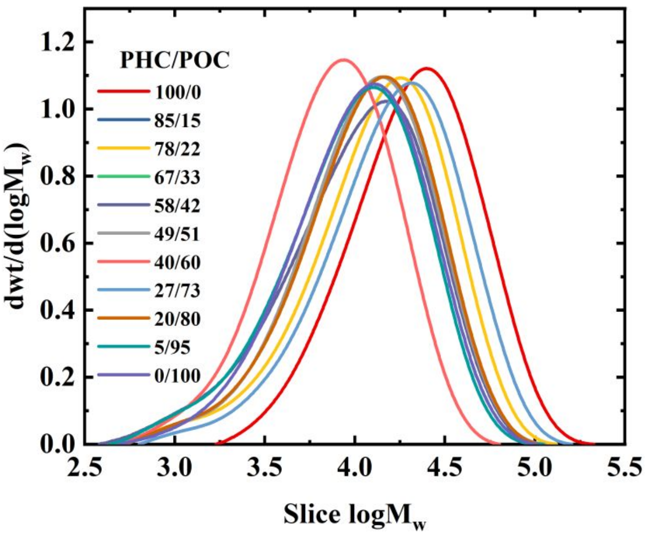

Figure S4. Monomodal curves of synthesized polymers, having different mol% of compositions.

Section S2. Thermal properties and morphologies

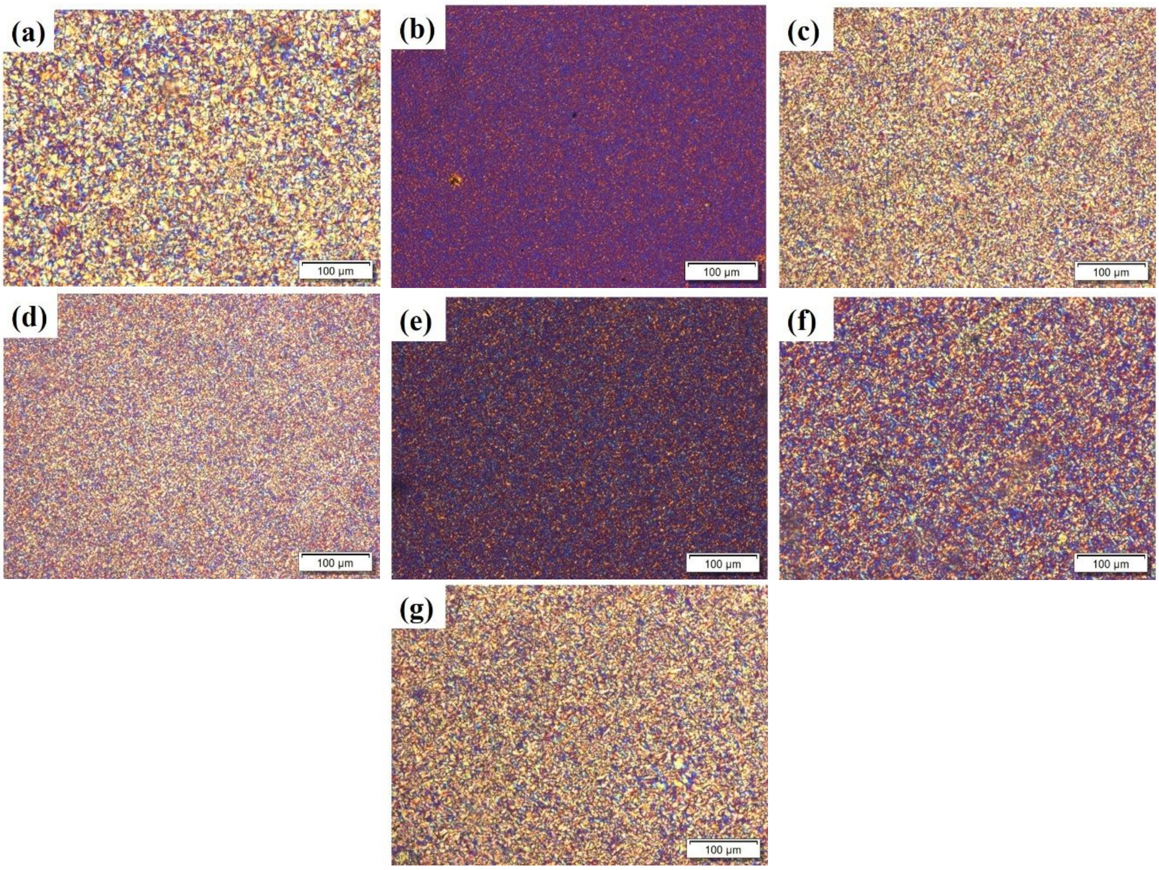

Figure S5. Polarized light optical micrographs for (a) PHC, (b) $PH_{78}O_{22}C$, (c) $PH_{67}O_{33}C$, (d) $PH_{49}O_{51}C$, (e) $PH_{20}O_{80}C$, (f) $PH_5O_{95}C$, and (g) POC taken at -40 ºC after cooling from the isotropic melt at 1 ºC/min.

The equilibrium melting enthalpy, $\Delta H_m°$, cannot be obtained from experiments directly, and the copolymers studied in this work had never been reported before. Therefore, the $\Delta H_m°$ was estimated with the group contribution theory of Van Krevelen.[1] The melting enthalpy of copolymers is mainly derived from the CH_2 group with a value of 4 kJ/mol, while the contribution from the –OCOO- group is supposed to be negligible. Hence, the $\Delta H_m°$ can be estimated by equation S1:

$$\Delta H_m^0 = \frac{\Delta H_{CH2} \cdot n_{CH2}}{M_i} \qquad \text{(S1)}$$

where ΔH_{CH2}=4 kJ/mol, n_{CH2} is the methylene units length, and M_i is the average molecular weight of copolymers.

The methylene length of copolymers contributed by two components in copolymer (i.e., PHC and POC) can be estimated based on the compositions by equation S2:

$$n_{CH2} = n_{HC} \times x_{PHC} + n_{OC} \times x_{POC} \qquad \text{(S2)}$$

where n_{HC} and n_{OC} are the methylene lengths of PHC and POC, with values of 6 and 8 respectively, and x_{PHC} and x_{POC} are the molar fraction of PHC and POC in copolymers, respectively.

Similarly, the average molecular weight could be calculated by equation S3:

$$M_i = M_{HC} \times x_{PHC} + M_{OC} \times x_{POC} \qquad \text{(S3)}$$

where M_{HC} and M_{OC} are the average molecular weight of HC and OC units, with values of 144 g/mol and 172 g/mol.

Table S1 shows the values employed in equation S1 and the calculated results.

Table S1. Employed values in equation S1, and final results.

Sample	ΔH_{CH2} (kJ/mol)	n_{CH2}		M_i (g/mol)		ΔH_m^0 (J/g)
		n_{PHC}	n_{POC}	M_{PHC}	M_{POC}	
PHC	4	6.00	--	144.0	--	167.0
$PH_{85}O_{15}C$	4	5.10	1.20	122.4	25.8	170.0
$PH_{78}O_{22}C$	4	4.68	1.76	112.3	37.8	171.6
$PH_{67}O_{33}C$	4	4.02	2.64	96.5	56.8	173.8
$PH_{58}O_{42}C$	4	3.48	3.36	83.5	72.2	175.7
$PH_{49}O_{51}C$	4	2.94	4.08	70.6	87.7	177.4
$PH_{40}O_{60}C$	4	2.40	4.80	57.6	103.2	179.1

$PH_{27}O_{73}C$	4	1.62	5.84	38.9	125.6	181.5
$PH_{20}O_{80}C$	4	1.20	6.40	28.8	137.6	182.7
$PH_{5}O_{95}C$	4	0.30	7.60	7.2	163.4	185.2
POC	4	--	8.00	--	172.0	186.0

Therefore, the crystallinity from DSC measurements, $X_{\mathrm{c,DSC}}$, could be estimated using equation S4:

$$X_{c,DSC} = \frac{\Delta H_m}{\Delta H_m^0} \times 100\% \quad \text{(S4)}$$

where ΔH_m is the melting enthalpy of samples obtained during the second heating process at 10 ºC/min (i.e., the melting of crystals formed during cooling crystallization from the isotropic melt). The $\Delta H_m°$ represents the equilibrium melting enthalpy shown in Table S1. The calculated crystallinities $X_{c,\mathrm{DSC}}$ for all materials are plots as a function of POC composition in Figure S6 and compared with the one estimated and used in the manuscript by WAXS measurements, $X_{c,\mathrm{WAXS}}$.

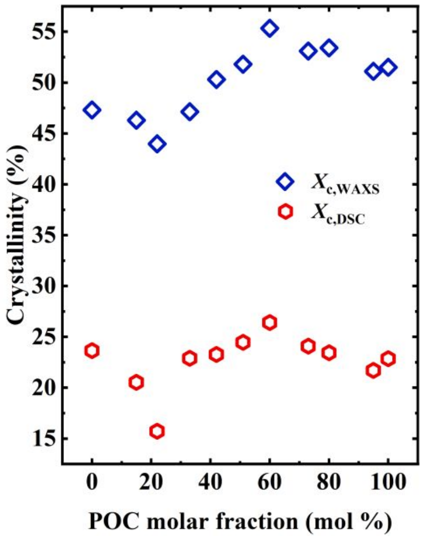

Figure S6. The crystallinities determined from (a) DSC melting enthalpy ($X_{c,\mathrm{DSC}}$) and (b) peak fitting of WAXS curves ($X_{c,\mathrm{WAXS}}$) as a function of POC content.

Section S3. WAXS analysis

The samples' crystallinity degree can be determined by conducting multiple peak-fitting analyses on the WAXS curves. Figure S7 gives an example of the peak fitting process for WAXS profiles. Using equation S5, the mass fraction of the crystalline phase can be calculated:

$$X_{c,\mathrm{WAXS}} = \frac{A_c}{A_c + A_a} \tag{S5}$$

where A_c represents the area of all diffraction peaks of crystalline, and A_a represents the area of the amorphous phase.

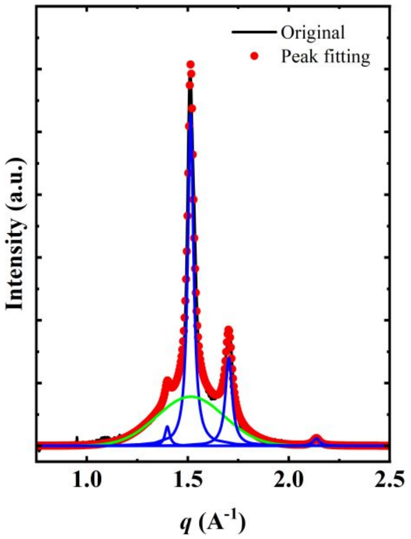

Figure S7. Example of multiple peak fitting analysis of WAXS profile of $PH_{20}O_{80}C$ copolymer obtained at -40 ºC to decompose the crystalline and amorphous phases fraction. The black curve represents the original data, and the red dots indicate fitting results. The blue sharp curves correspond to those crystalline phases, while the green broad peak corresponds to the amorphous phase.

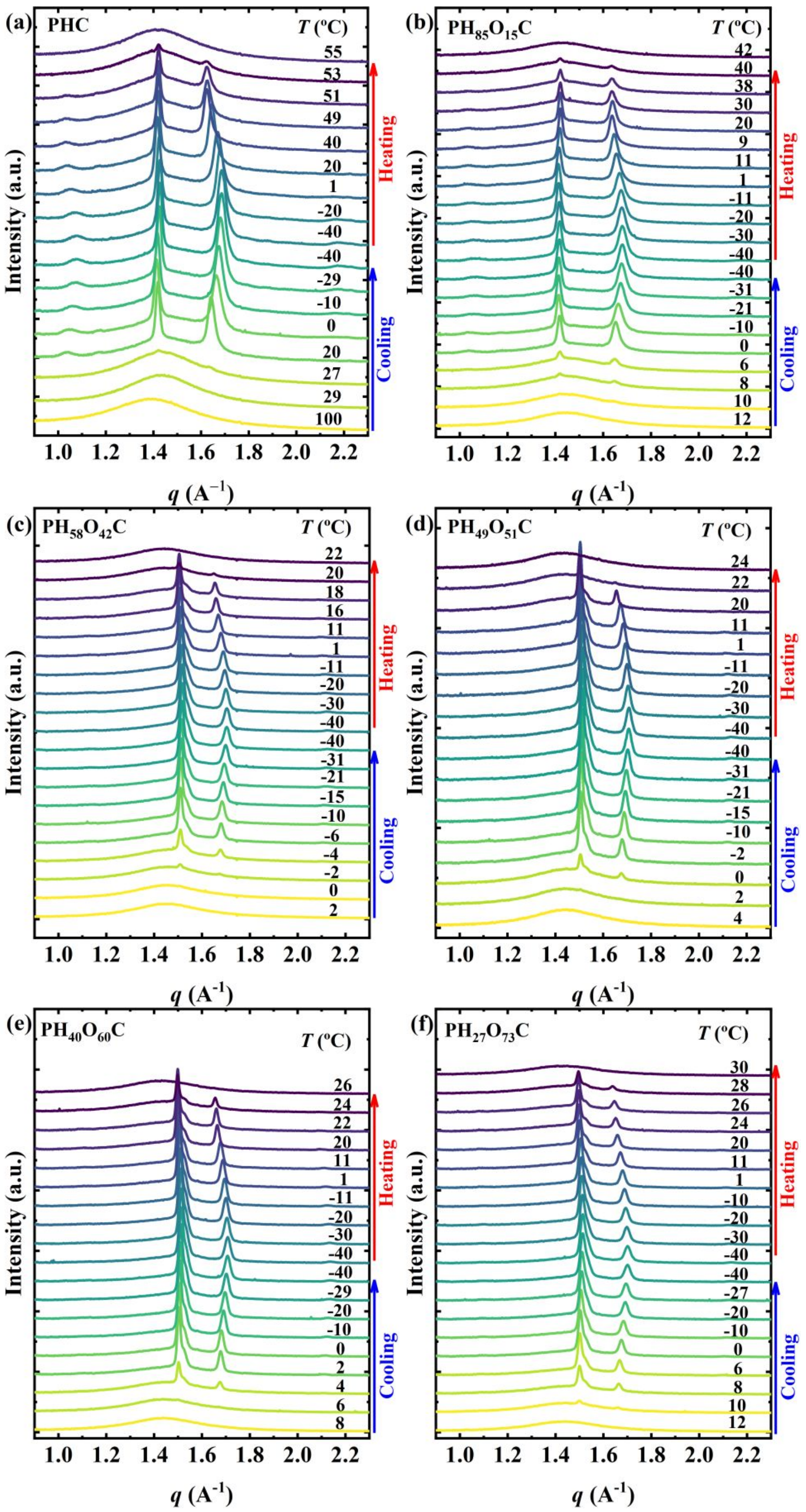

(a) PHC
T (ºC)
55 53 51 49 40 20 1 -20 -40 -40 -29 -10 0 20 27 29 100
Heating
Cooling
(b) PH$_{85}$O$_{15}$C
T (ºC)
42 40 38 30 20 9 11 1 -11 -20 -30 -40 -40 -31 -21 -10 0 6 8 10 12
(c) PH$_{58}$O$_{42}$C
T (ºC)
22 20 18 16 11 1 -11 -20 -30 -40 -40 -31 -21 -15 -10 -6 -4 -2 0 2
(d) PH$_{49}$O$_{51}$C
T (ºC)
24 22 20 11 1 -11 -20 -30 -40 -40 -31 -21 -15 -10 -2 0 2 4
(e) PH$_{40}$O$_{60}$C
T (ºC)
26 24 22 20 11 1 -11 -20 -30 -40 -40 -29 -20 -10 0 2 4 6 8
(f) PH$_{27}$O$_{73}$C
T (ºC)
30 28 26 24 20 11 1 -10 -20 -30 -40 -40 -27 -20 -10 0 6 8 10 12
Intensity (a.u.)
q (A^{-1})
1.0 1.2 1.4 1.6 1.8 2.0 2.2

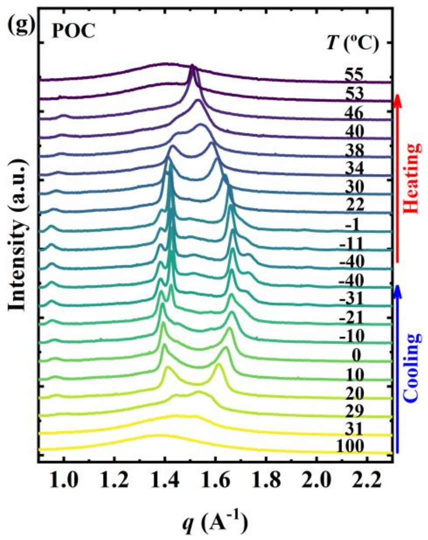

Figure S8. Temperature-dependent WAXS diffractograms acquired during cooling and heating at 10 ºC/min for (a) PHC, (b) $PH_{85}O_{15}C$, (c) $PH_{58}O_{42}C$, (d) $PH_{49}O_{51}C$, (e) $PH_{40}O_{60}C$, (f) $PH_{27}O_{73}C$, and (g) POC.

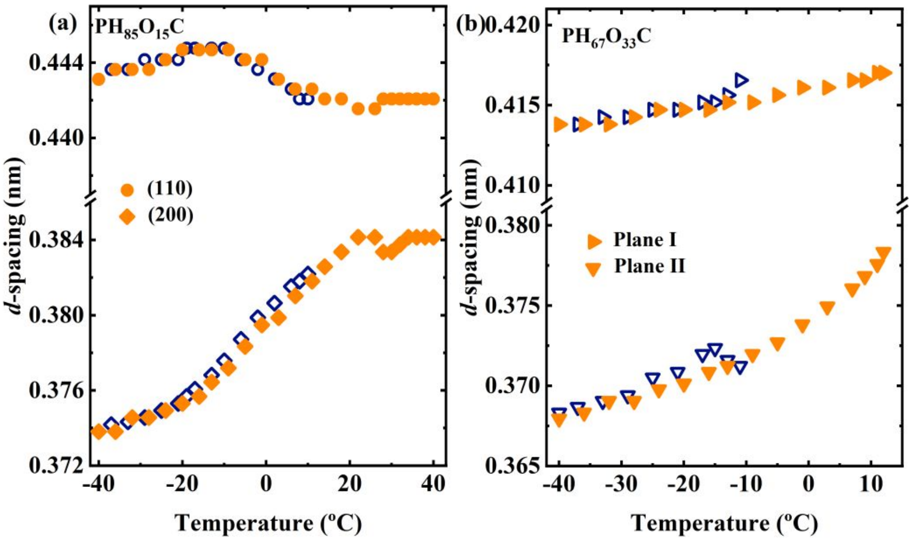

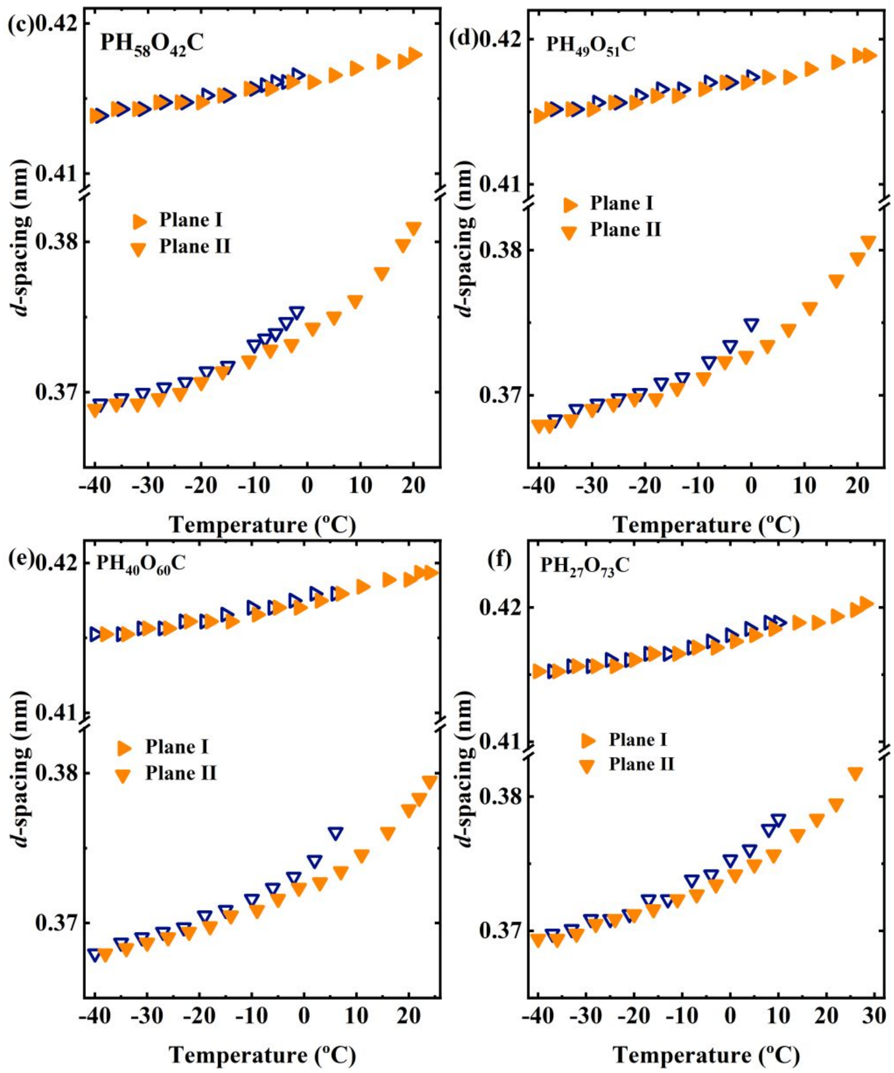

Figure S9. *d*-spacing evolution for (a) $PH_{85}O_{15}C$, (b) $PH_{67}O_{33}C$, (c) $PH_{58}O_{42}C$, (d) $PH_{49}O_{51}C$, (e) $PH_{40}O_{60}C$, and (f) $PH_{27}O_{73}C$. The blue open points represent the cooling process, while the orange solid points correspond to the heating process.

Section S4. SAXS analysis

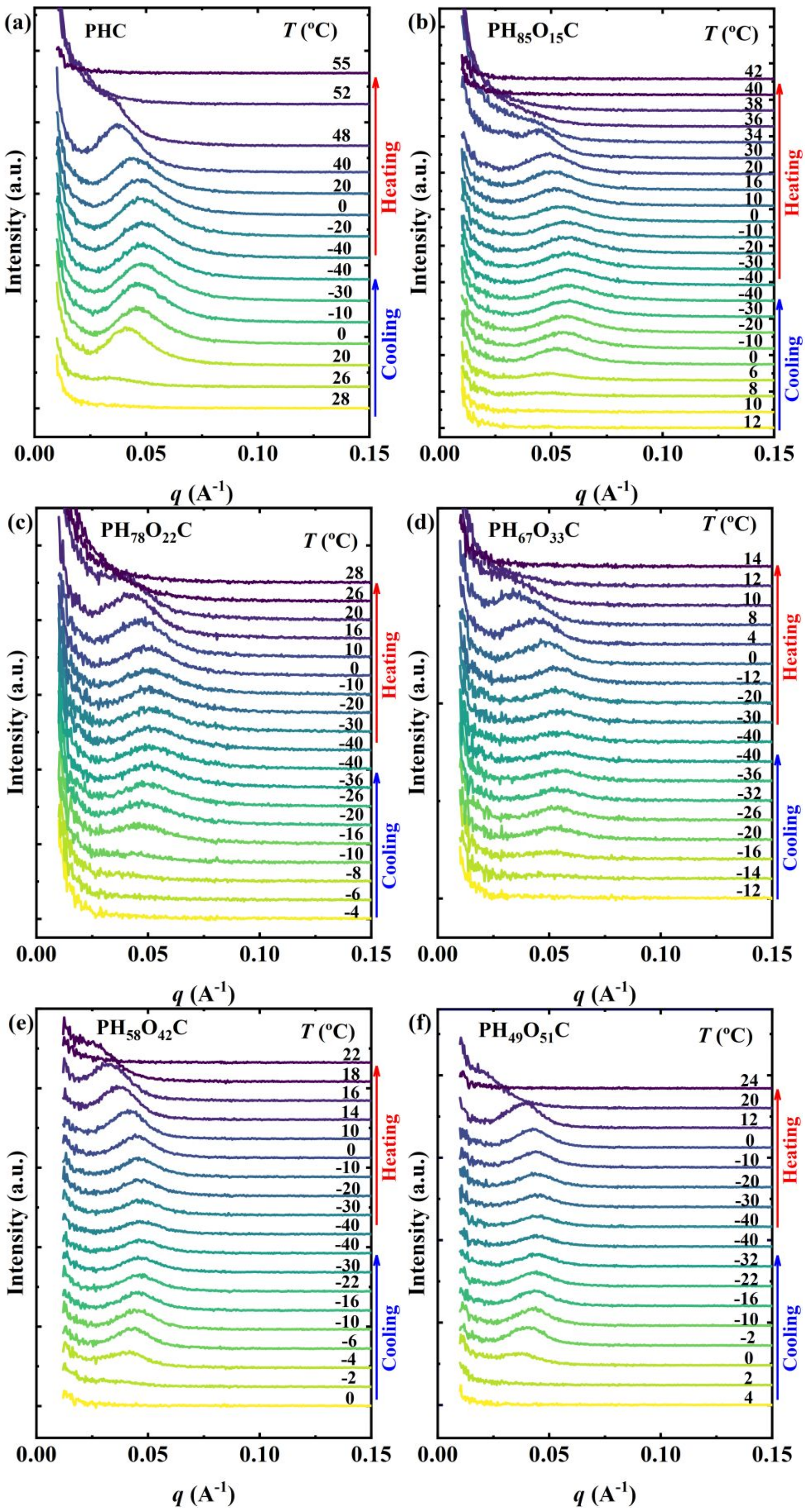

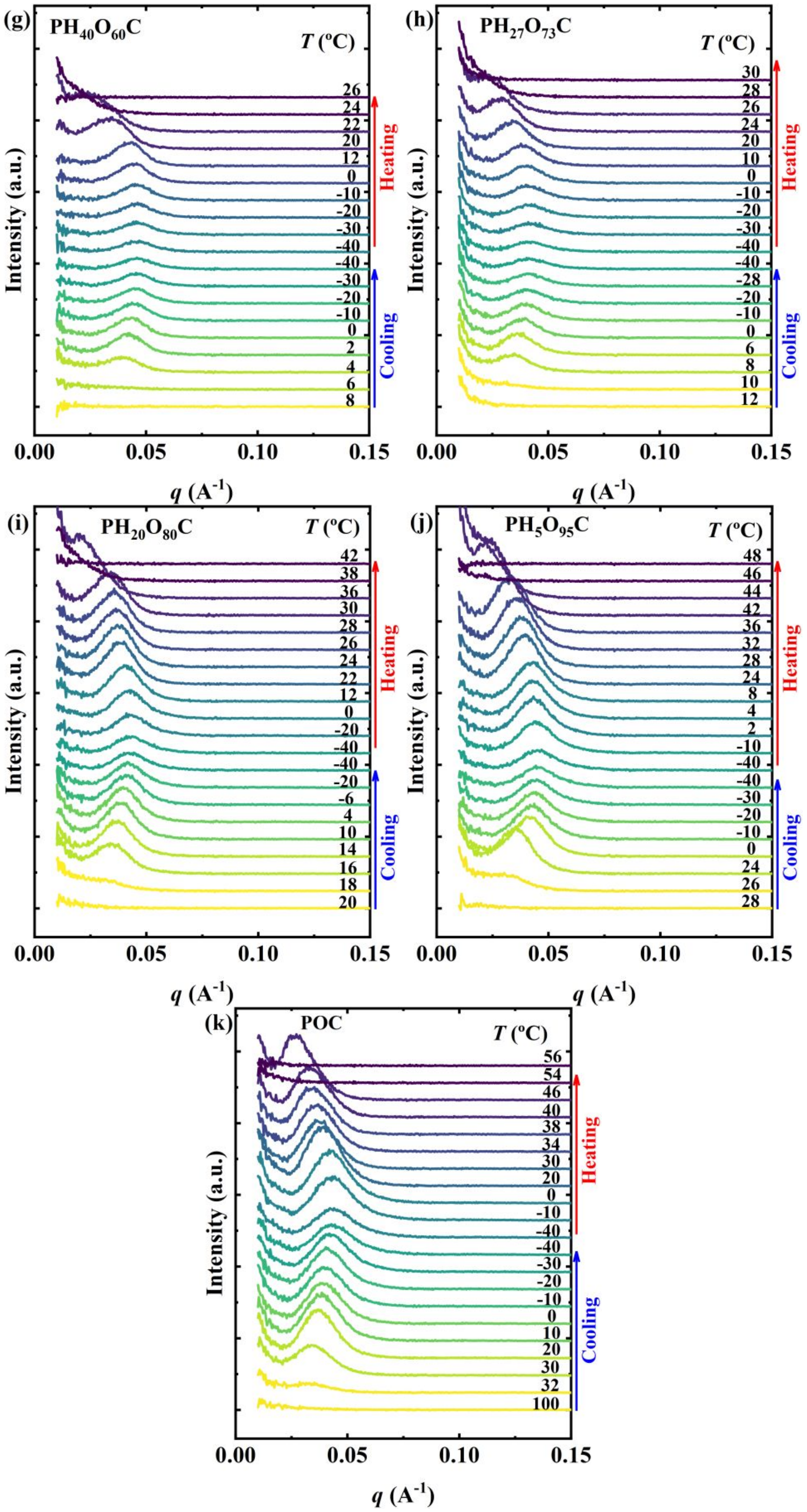

Figure S10. Temperature-dependent SAXS profiles recorded during the cooling and heating process at 10 ºC/min for studied materials in this work: (a) PHC, (b) $PH_{85}O_{15}C$, (c) $PH_{78}O_{22}C$,

(d) $PH_{67}O_{33}C$, (e) $PH_{58}O_{42}C$, (f) $PH_{49}O_{51}C$, (g) $PH_{40}O_{60}C$, (h) $PH_{27}O_{73}C$, (i) $PH_{20}O_{80}C$, (j) $PH_{5}O_{95}C$, and (k) POC.

Section S5. FTIR analysis

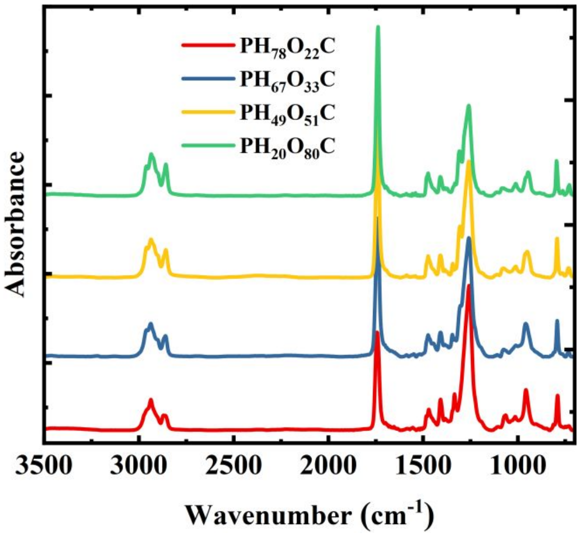

Figure S11. FTIR spectra obtained at -40 ºC for the typical copolymers of $PH_{78}O_{22}C$, $PH_{67}O_{33}C$, $PH_{49}O_{51}C$, and $PH_{20}O_{80}C$.

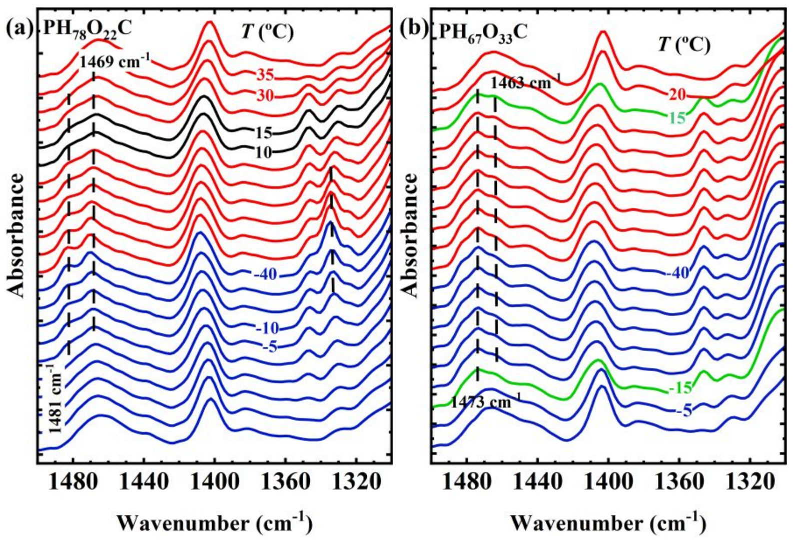

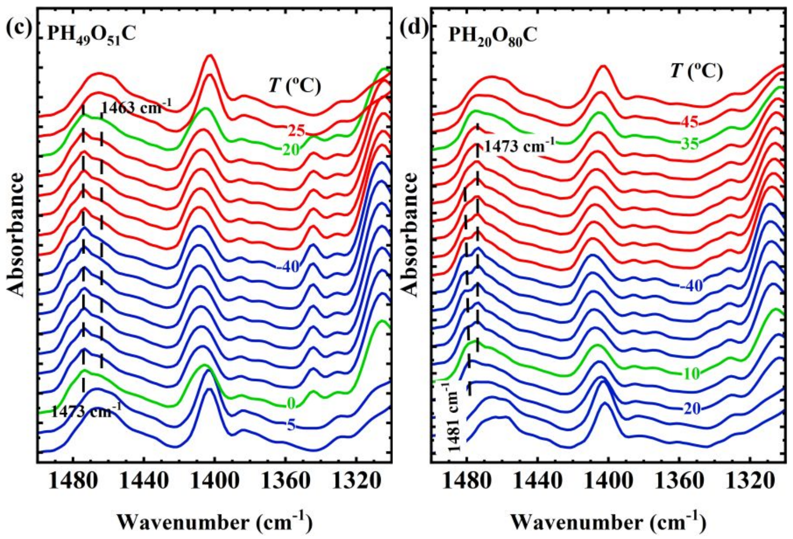

Figure S12. Variable temperature FTIR spectra in the range of 1500-1300 cm^{-1} for (a) $PH_{78}O_{22}C$, (b) $PH_{67}O_{33}C$, (c) $PH_{49}O_{51}C$, and (d) $PH_{20}O_{80}C$. The blue curves indicate the cooling process, while the red curves represent the subsequent heating process. The green curves indicate the appearance and disappearance of the new phase.

Section S6. Understanding the crystallization behavior of the new crystalline phase.

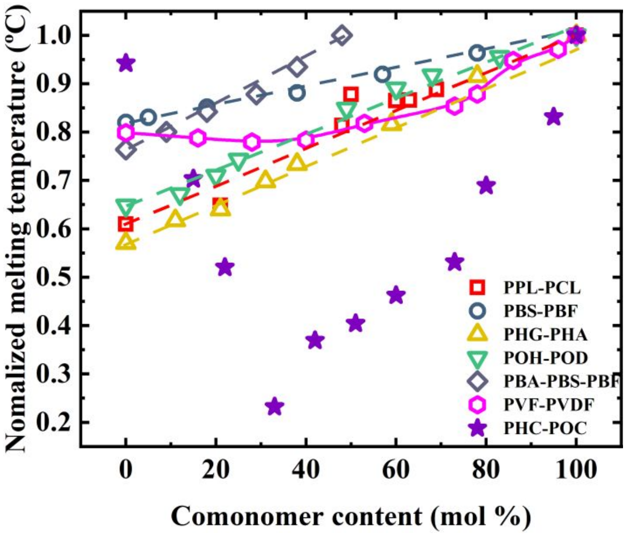

Figure S13. Normalized melting temperature as a function of comonomer content for selected isomorphic copolymers. The open points were extracted from literature,[2-7] and the

solid points correspond to the copolymers studied in this work. PPL-PCL: poly(□-pentadecalactone)-*ran*-poly(ε-caprolactone); PBS-PBF: poly (butylene succinate-*ran*-butylene fumarate); PHG-PHA: poly (hexamethylene gluarate-*ran*-hexamethylene azelate); POH-POD: poly (oxyhexamethylene-*ran*-oxydodecamethylene); PBA-PBS-PBF: poly (butylene adipate-*ran*-butylene succinate-*ran*-butylene fumarate); PVF-PVDF: poly (vinyl fluoride-co-vinylidene fluoride); PHC-POC: poly (hexamethylene-*ran*-octamethylene carbonate).

REFERENCES

1. Van Krevelen, D. W., Chapter 5 - Calorimetric Properties. In *Properties of Polymers (Third Edition)*, Van Krevelen, D. W., Ed. Elsevier: Amsterdan, 1997; pp 109-127.
2. Ceccorulli, G.; Scandola, M.; Kumar, A.; Kalra, B.; Gross, R. A., Cocrystallization of Random Copolymers of ω-Pentadecalactone and ε-Caprolactone Synthesized by Lipase Catalysis. *Biomacromolecules* **2005,** *6* (2), 902-907.
3. Ye, H.-M.; Wang, R.-D.; Liu, J.; Xu, J.; Guo, B.-H., Isomorphism in Poly(butylene succinate-co-butylene fumarate) and Its Application as Polymeric Nucleating Agent for Poly(butylene succinate). *Macromolecules* **2012,** *45* (14), 5667-5675.
4. Yu, Y.; Sang, L.; Wei, Z.; Leng, X.; Li, Y., Unique isodimorphism and isomorphism behaviors of even-odd poly(hexamethylene dicarboxylate) aliphatic copolyesters. *Polymer* **2017,** *115*, 106-117.
5. Basterretxea, A.; Gabirondo, E.; Flores, I.; Etxeberria, A.; Gonzalez, A.; Müller, A. J.; Mecerreyes, D.; Coulembier, O.; Sardon, H., Isomorphic Polyoxyalkylene Copolyethers Obtained by Copolymerization of Aliphatic Diols. *Macromolecules* **2019,** *52* (9), 3506-3515.
6. Wei, X.-W.; Huang, G.; Wang, J.; Meng, X.; Zhou, Q.; Ye, H.-M., Tailoring Crystallization of Random Terpolyester: Combination of Isodimorphism and Isomorphism. *Macromolecules* **2020,** *53* (20), 8918-8927.
7. Natta, G.; Allegra, G.; Bassi, I. W.; Sianesi, D.; Caporiccio, G.; Torti, E., Isomorphism phenomena in systems containing fluorinated polymers and in new fluorinated copolymers. *J. Polym. Sci., Part A: Gen. Pap.* **1965,** *3* (12), 4263-4278.
